# Quantification of lactate from various metabolic pathways and quantification issues of lactate isotopologues and isotopmers

**DOI:** 10.1038/s41598-017-08277-3

**Published:** 2017-08-16

**Authors:** Wei Zhang, Cheng Guo, Kezhi Jiang, Minfeng Ying, Xun Hu

**Affiliations:** 1 Cancer Institute (a Key Laboratory For Cancer Prevention & Intervention, China National Ministry of Education), The Second Affiliated Hospital, Zhejiang University School of Medicine, 88 Jiefang Road, Hangzhou, China; 2 Key Laboratory of Organosilicon Chemistry and Material Technology, Hangzhou Normal University, Hangzhou, China

## Abstract

^13^C-labeled glucose combined with chromatography and mass spectrometry enables us to decipher the percentage of lactate generated from various metabolic pathways. We showed that lactate derived from glycolysis, pentose phosphate pathway, Krebs cycle, and other sources accounted for 82–90%, 6.0–11%, 0.67–1.8% and 1.5–7.9%, respectively, depending on different types of cells. When using glucose isotopomers ([1-^13^C]-, [3-^13^C]-, [4-^13^C]-, and [6-^13^C]glucose) or isotopologues ([1,2-^13^C_2_]- and [1,2,3-^13^C_3_]glucose) for tracing, the ratio of lactate derived from glucose carbon 1, 2, 3 over 4, 5, 6 via glycolysis varied significantly, ranging from 1.6 (traced with [1,2-^13^C_2_]glucose) to 0.85 (traced with [6-^13^C]glucose), but the theoretical ratio should be 1. The odd results might be caused by intramolecular ^13^C, which may significantly affect lactate fragmentation under tandem mass spectrometry condition, leading to erroneous quantification. Indeed, the fragmentation efficiency of [U-^13^C]lactate, [2,3-^13^C]lactate, and [3-^13^C]lactate were 1.4, 1.5 and 1.2 folds higher than lactate, respectively, but [1-^13^C]lactate was similar to lactate, suggesting that carbon-13 at different positions could differentially influence lactate fragmentation. This observed phenomenon was inconsistent with the data based on theoretical calculation, according to which activation energies for all lactate isotopomers and isotopologues are nearly identical. The inconsistency suggested a need for further investigation. Our study suggests that calibration is required for quantifying metabolite isotopolugues and isotopomers.

## Introduction

Cancer cells convert most incoming glucose to lactate, a metabolic hallmark called Warburg effect^1, 2^. Lactate and proton are important for cancer cells to survive through harsh conditions. We recently demonstrated that lactate and proton together switched cancer cells from Warburg effect to an economical metabolic mode with negligible or no net generation of lactate^3^ and with 90% ATP from oxidative phosphorylation^4^. Moreover, lactate and proton together prevented cancer cells from glucose deprivation-induced death^5^. The findings suggested that targeting intratumoral lactic acidosis might be considered as a therapeutic target. Indeed, our clinical study demonstrated a remarkable effect of bicarbonate on local control of hepatocellular carcinoma^6^.

Many other investigators have independently reported the significance of intratumoral lactic acidosis in tumor biology. Clinical studies showed that high level of lactate was a strong prognostic indicator of increased metastasis and poor overall survival^7–13^. Gillies and Gatenby group demonstrated that systematic and tumor pHe alkalization could inhibit carcinogenesis, tumor invasion and metastasis, and they also provided integrated models that can predict the safety and efficacy of buffer therapy to raise tumor pHe^14–16^ and related theoretical work^17, 18^. Furthermore, lactic acidosis exhibited multifaceted roles in skewing macrophages^19^, inhibiting the function of cytotoxic T cells^20^, altering cancer cell metabolism^21, 22^, inducing chromosomal instability^23^, and promoting tumor angiogenesis^7, 24^.

Hence, lactate generation is an interesting topic in cancer metabolic research. Glucose^25^ is the main sources of lactate generation in cancer cell metabolism. However, the percentage of lactate generated from glucose through various metabolic pathways (e.g., glucose carbons metabolism through glycolysis, pentose phosphate pathway, and Krebs cycle) is not known. The purpose of this study is to investigate this issue.

## Materials and Methods

### Cell culture and sample preparation

4T1 and Hela cells are maintained in RPIM-1640 (Life Technologies) supplemented with 10% fetal bovine serum (FBS). 2 × 10^4^ 4T1 (or Hela) cells are seeded into 96-well plate to allow attachment overnight in a humidified CO_2_ incubator. The culture medium was then replaced with serum free RPMI 1640 with 6 mM [1,2-^13^C_2_]glucose ([1-^13^C]-, [3-^13^C]-, [4-^13^C]-, [6-^13^C]-, [1,2,3-^13^C_3_]- or [U-^13^C]glucose) and incubated for another 12 hours. Cell culture supernatant was collected by centrifugation and stored at −20 °C. 4 × 10^6^ K562 cells were seeded in serum free RPIM 1640 with 6 mM [1,2-^13^C_2_]glucose ([1-^13^C]-, [3-^13^C]-, [4-^13^C]-, [6-^13^C]-, [1,2,3-^13^C_3_]- or [U-^13^C]glucose) into 96-well cell culture plate. After 12 hours incubation in a humidified CO_2_ incubator, cell culture supernatant was collected by centrifugation and stored at −20 °C.

Thymocytes were prepared from thymus of 4-week old female ICR mice purchased from SLRC laboratory animal (Shang Hai, China). Thymus glands were squeezed by syringe inner plunger and the suspension was filtered through 100 μm cell strainer. Thymocytes were incubated at a density of 2 × 10^7^ cells/ml in serum free RPIM 1640 with 6 mM [1,2-^13^C_2_]glucose ([1-^13^C]-, [3-^13^C]-, [4-^13^C]-, [6-^13^C]-, [1,2,3-^13^C_3_]- or [U-^13^C]glucose) for 12 hours in a humidified CO_2_ incubator, and supernatant was collected by centrifugation and stored at −20 °C.

Prior to analysis by LC-MS/MS, 10 μl sample was diluted by 190 μl acetonitrile, and cleaned by centrifugation at 10000 g for 30 min at 4 °C, supernatant was collected and stored at −20 °C.

### Enzymatic determination of lactate

The concentration of lactate is measured according to previously reported method with modification^26^. Briefly, 10 μl cell culture supernatant was added into 590 μl assay solution containing 2 mM nicotinamide adenine dinucleotide (NAD), 10 U lactate dehydrogenase (LDH), in reaction buffer (200 mM glycine and 170 mM hydrazine, pH 9.2), mixed well, incubated at 37 °C for 30 min, and read at 340 nm against a water blank with a ultraviolet spectrophotometer reader.

### Analysis of Lactate by LC-MS/MS

Lactate isotopomers and isotopologues of the cell culture supernatant were measured by liquid chromatography-tandem mass spectrometry (LC-MS/MS). LC was performed on a Waters ACQUITY UPLC system employing a ACQUITY BEH Amide column, 2.1 × 100 mm, 1.7 μm. Eluent A was 95% acetonitrile and 5% aqueous solution of 20 mM ammonium acetate (pH 9.0), whereas eluent B was 50% acetonitrile and 50% aqueous solution of 20 mM ammonium acetate (pH 9.0). The flow rate was set at 0.6 ml/min, injection volume is 5.0 μl, and the column was kept at 50 ^o^C. The optimized gradient conditions were adopted from Waters ACQUITY UPLC BEH Amide column application notebook with a minor modification as follows: 0-0.4 min hold for 99.9% eluent A, 0.4–0.5 min from 99.9% −60% eluent A, 0.5–2 min from 60% −30% eluent A, 2–2.1 min from 30% −99.9% eluent A, and hold for 10 min. The retention time of lactate and lactate isotopomers and isotopologues was 1.29 min.

The MS detection was performed on a 4000 QTRAP mass spectrometer (AB SCIEX, Foster City, CA, USA) equipped with an ESI ion source (Turbospray) operated in negative ion mode. Instrument control, data acquisition, and processing were performed using the Analyst 1.5.2 software. Firstly, collision-induced dissociation (CID) experiment of lactic acid standard was performed in product ion scan mode and the spectrum was illustrated in Supplementary Fig. S1A. The dissociation mechanism was proposed as illustrated in Supplementary Fig. S1B. Then MS/MS data were acquired in the multiple reaction monitoring (MRM) mode. The transition between precursor ion and the most abundant product ion (*m*/*z* 89.0 > 43.0) was monitored for quantitative determination. The ion transition *m*/*z* 89.0 > 71.0 was monitored for qualitative analysis to confirm the identity of lactic acid in samples. To determine the percentage of ^13^C-labeled lactate derived from ^13^C-labeled glucose through glycolysis and pentose phosphate pathway, the following ion transitions were monitored to determine the distribution of ^13^C-labeled lactate: *m*/*z* 90.0 > 43.0, [1-^13^C]lactate; *m*/*z* 90.0 > 44.0, [2-^13^C]- and [3-^13^C]lactate; *m*/*z* 91.0 > 44.0, [1,2-^13^C_2_]- and [1,3-^13^C_2_]lactate; *m*/*z* 91.0 > 45.0, [2,3-^13^C_2_]lactate; and *m*/*z* 92.0 > 45.0, [U-^13^C]lactate.

To increase sensitivity, the ion source temperature (TEM) was set at 500 °C, and the ion spray voltage (IS) was set at −4.5 kV. Ion source gas 1 (GS1) and ion source gas 2 (GS2) used as the nebulizing and drying gases were set at 50 and 40 psi, respectively. Curtain gas (CUR) was set at 40 psi. The optimized MS conditions used for the analysis of the target analytes were shown in Supplementary Table S1.

### Eliminate peak area of natural ^13^C-labled lactate

Chemical structure of lactate and the numbering of its carbon, hydrogen and oxygen atom are shown in Supplementary Fig. S2, according to isotopic abundances of carbon, hydrogen, oxygen elements (Supplementary Table S2) and proposed fragmentation mechanism of lactate (Supplementary Fig. S1B), the relative percentage of each natural ^13^C-labled lactate (*m*/*z* 90 > 44, 90 > 43, 91 > 45, 91 > 44 and 92 > 45) could be calculated (Supplementary Tables S3–S4). We used formula (1) to exclude the peak area of lactate isotopomers and isotopologues that from nature. Let Χ be the peak area of ^13^C-labled lactate produced by cells, P_L_ be the measured peak area of ^13^C-labled lactate which includes ^13^C-labled lactate existed in nature and that produced by cells, P_U_ be the peak area of unlabeled lactate, R be the corresponding relative percentage of each natural ^13^C-labled lactate.

1 $${\rm{{\rm X}}}={{\rm{P}}}_{{\rm{L}}}-{{\rm{P}}}_{{\rm{U}}}\times {\rm{R}}$$

### Theoretical calculations of the activation energy for fragmentation of lactate isotopomers and isotopologues

All theoretical calculations were performed by using the density functional theory (DFT) method at the B3LYP/6-31 G(d) level of theory in the Gaussian 03 program^27^. The optimized structures for the precursor ions, intermediates and products were identified as a true minimum in energy by the absence of imaginary frequencies. Transition states, on the other hand, were identified by the presence of one single imaginary vibration frequency with the normal vibrational mode, and further confirmed by the intrinsic reaction coordinates (IRC) analysis. The energies discussed here are the sum of electronic and thermal enthalpies. The DFT optimized structures were shown by Gauss View (version 3.07) software to give higher quality images of these structures.

### Statistical analysis

All the statistical analyses were performed using SPSS statistics 19.0 software (IBM, Armonk, NY, USA). T-test was applied to evaluate the differences of peak areas ratio of different ^13^C-lableing lactate.

## Results and Discussion

### The rationale for quantification of lactate isotopomers and isotopologues

In this study, we used 7 glucose isotopomers ([1-^13^C]-, [3-^13^C]-, [4-^13^C]-, and [6-^13^C]glucose) or isotopologues ([1,2-^13^C_2_]-, [1,2,3-^13^C_3_]- and [U-^13^C]glucose) to trace lactate. Accordingly, 7 lactate isotopomers or isotopologues ([1-^13^C]-, [2-^13^C]-, [3-^13^C]-, [1,2-^13^C_2_]-, [1,3-^13^C_2_]-, [2,3-^13^C_2_]-, and [U-^13^C]lactate) would be generated.

The percentage of each lactate isotopomers or isotopologues was quantified by tandem mass spectrometry. Under MRM mode of mass spectrometer with triple quadrupole linear ion trap (QTRAP) analyzer, the precursor ion *m*/*z* 89.0 was isolated in the first quadrupole (Q1), subsequently, the ion dissociated under collisional activation using collision gas such as nitrogen in Q2, and the fragment ions were isolated in Q3, finally, the ions were detected by the detector, as described in Supplementary Fig. S3.

As illustrated in Supplementary Fig. S1A, two fragment ions at *m*/*z* 71.0 and 43.0 were produced in the dissociation of unlabeled lactate at *m*/*z* 89.0. The transition between precursor ion and the most abundant product ion (*m*/*z* 89.0 > 43.0) was monitored for quantitative determination. And the ion transition *m*/*z* 89.0 > 71.0 was monitored for qualitative analysis to confirm the identity of lactate in samples. For ^13^C-labeled lactate derived from ^13^C-labeled glucose through glycolysis and pentose phosphate pathway, the following ion transitions were monitored to determine the distribution of ^13^C-labeled lactate: *m*/*z* 90.0 > 43.0, [1-^13^C]lactate; *m*/*z* 90.0 > 44.0, [2-^13^C]- and [3-^13^C]lactate; *m*/*z* 91.0 > 44.0, [1,2-^13^C_2_]- and [1,3-^13^C_2_]lactate; *m*/*z* 91.0 > 45.0, [2,3-^13^C_2_]lactate; and *m*/*z* 92.0 > 45.0, [U-^13^C]lactate.

Representative chromatograms of lactate standard and major lactate species generated in cells were shown in Supplementary Fig. 4; the sum of their percentages was more than 98% of total lactate. And the retention time of lactate and each ^13^C-labeled lactate generated in cells is identical to that of lactate standard. The relative standard deviation (RSD) in the peak area is ≤10% (n = 6) for lactate and each ^13^C-labeled lactate mentioned in Supplementary Fig. 4. It can be clearly seen that lactate and each ^13^C-labeled lactate generated in cells can be detected with good peak shapes, and the signal to noise ratios is above ten. And lactate isotopomers and isotopologues can be separated from each other based on *m*/*z* value.

According to the established principle, ^13^C, which only adding a neutron into nucleus, should not affect chemical bonding, hence should not affect molecule fragmentation both quality and quantity under tandem mass spectrometry condition, i.e., the fragmentation of lactate with or without ^13^C should be exactly the same.

### A time course to determine the steady-state generation of lactate isotopologues or isotopomers

In order to exclude the interference of exogenous lactate, we used serum free RPMI-1640 to culture cells, as serum contains appreciable amount of lactate, which may interfere with the quantification of lactate isotopologues. Nevertheless, the condition of serum-free medium could be considered as a stress for cancer cells, which might interfere with the cell metabolism. We compared the percentage of lactate isotopologues generated by cells in the presence or absence of serum. The results were summarized in Supplementary Table S6, showing no significant difference between cultures with or without serum. Thus, we used serum free RPMI-1640 to culture cells in this study with the culture time within 12 hours.

**Figure 1:**
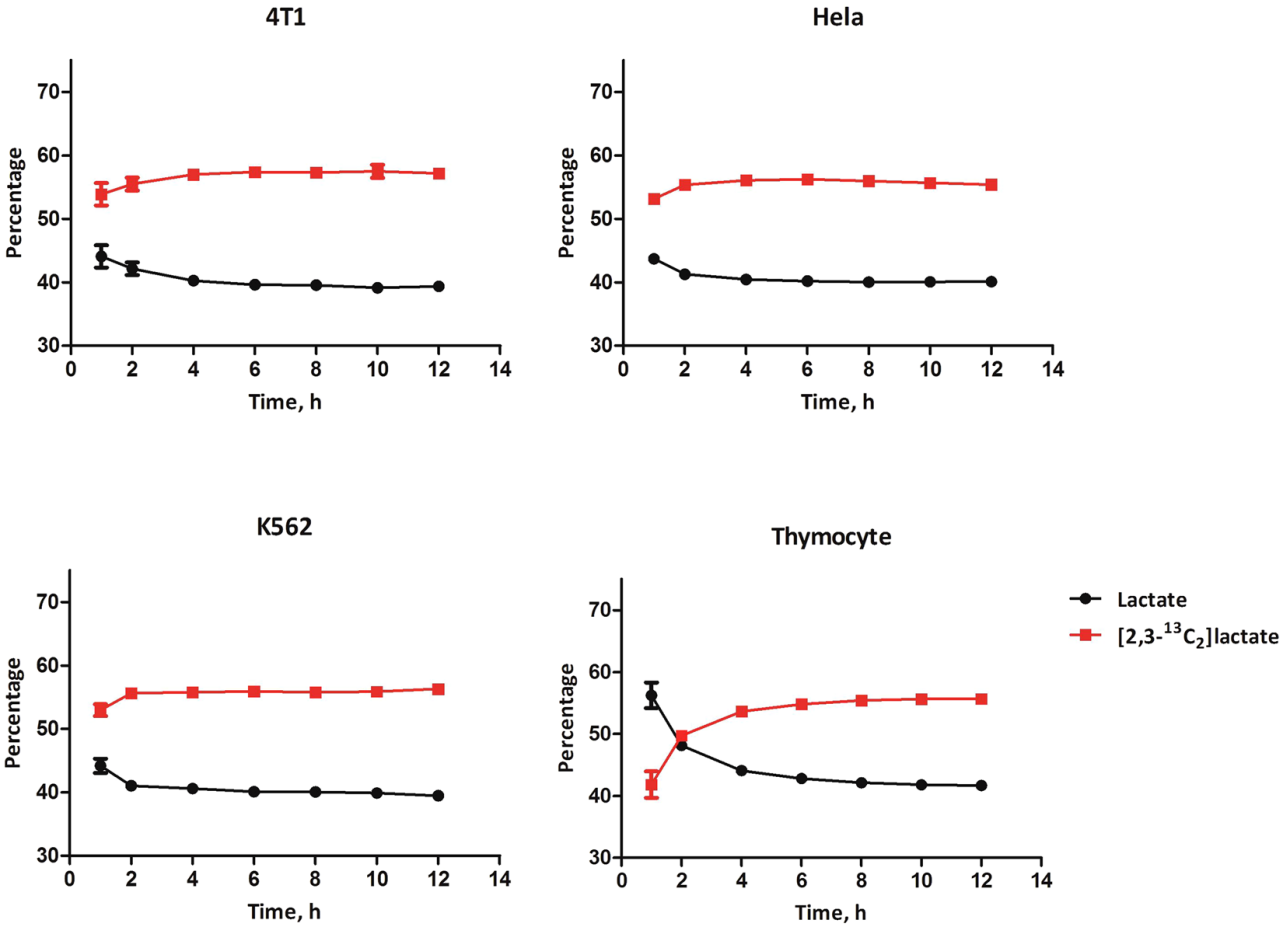
A time course of generation of [2,3-^13^C_2_]lactate (from glucose carbon 1, 2, 3) and lactate (from glucose carbon 4, 5, 6) (n = 12) from 4T1, Hela, K562 and thymocyte. Cells were incubated in serum free RPMI-1640 containing 6 mM [1,2-^13^C_2_]glucose and supernatant was collected for LC-MS analysis as described in Materials and Methods. Data were mean ± SD, n = 12, from 2 independent experiments.

We performed a time-course experiment and observed that a steady-state generation of lactate isotopologues was attained 4 hours after incubation and maintained thereafter (Fig. 1; Supplementary Table S5). Based on the time-course experiment, all the experiments performed below were carried out using serum free RPMI-1640, with an incubation time of 12 hours.

### Determination of lactate from glucose and other sources

**Figure 2:**
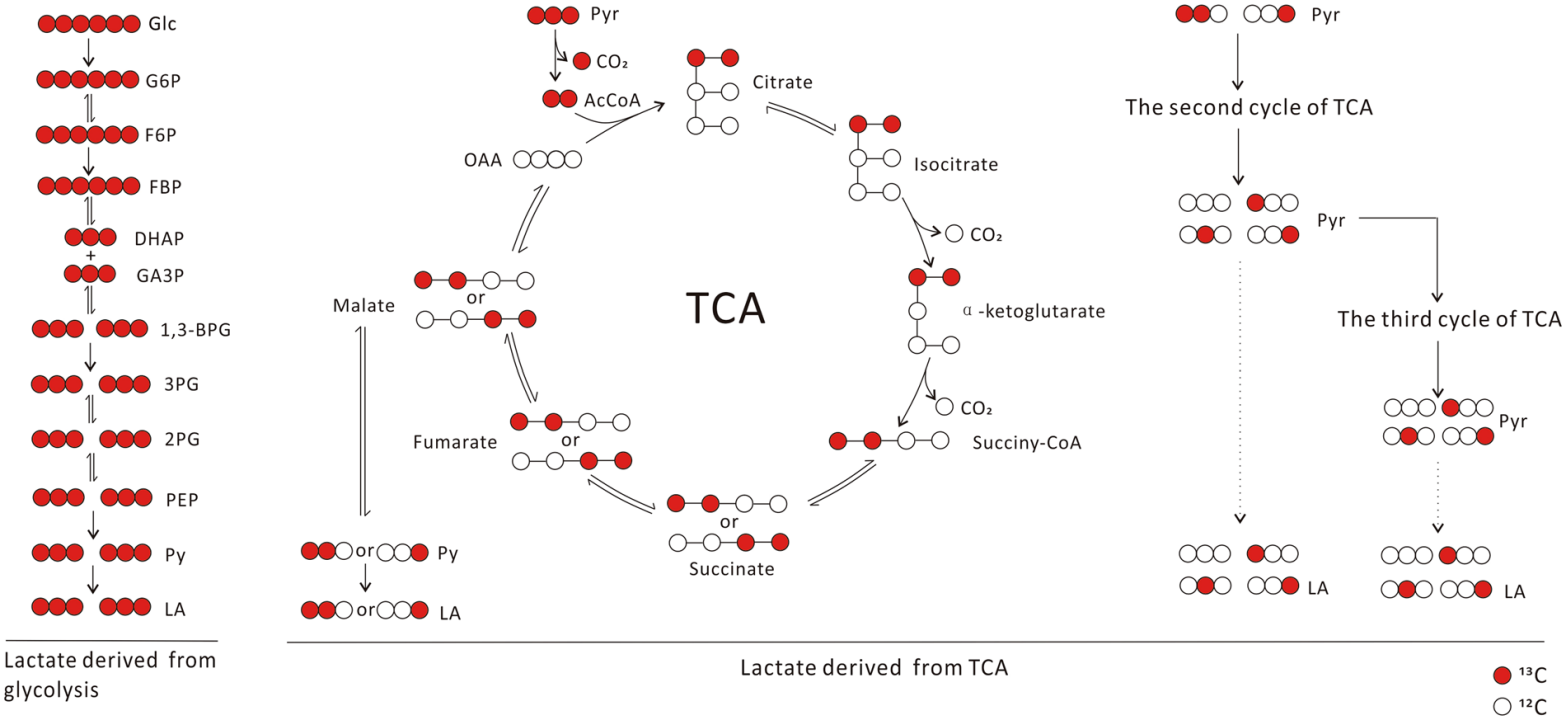
The metabolic pathways that generate various lactate isotopomers and isotopologues in 4T1 cells, traced by [U-^13^C] glucose. Besides [U-^13^C]lactate, there are trace amount of other lactate isotopologues, which could be generated from [U-^13^C]pyruvate through tricarboxylic cycle to produce [1,2-^13^C_2_]malate and [3,4-^13^C_2_]malate, which, via catalysis of malic enzyme, generate [1,2-^13^C_2_]pyruvate and [3-^13^C]pyruvate, which can convert to corresponding lactate. [1,2-^13^C_2_]pyruvate and [3-^13^C]pyruvate can enter Krebs cycle, leading to generation of other lactate isotopomers.

We used [U-^13^C]glucose to trace lactate. Glucose-derived lactate constituted about 97% of total lactate (Table 1), other source-derived lactate constituted about 2%, and lactate derived from mixed carbon sources (glucose and other sources) was less than 1%, which was most probably derived from Krebs cycle (Fig. 2). The percentage of lactate isotopologues generated from 4T1, Hela, and K562 cancer cells were comparable with each other. In mouse thymocytes, lactate generated from glucose, mixed carbon sources, and other sources constituted about 90%, 2%, and 8%, respectively.

**Table 1:** The percentage of lactate isotopomers and isotopologues (mean ± SD, n = 12) generated by 4T1, Hela, K562, and thymocytes, traced by [U-^13^C]glucose.

	**Lactate derived from [U-** ^**13**^ **C]glucose**	**Lactate derived from mixed carbon sources**				**Lactate derived from other sources**	
	**[U-** ^**13**^ **C]lactate**	**[2-** ^**13**^ **C]- or [3-** ^**13**^ **C]lactate**	**[1**,**2-** ^**13**^ **C**_**2**_ **]- or [1**,**3-** ^**13**^ **C**_**2**_ **]lactate**	**[1-** ^**13**^ **C]lactate**	**[2**,**3-** ^**13**^ **C**_**2**_ **]lactate**	**lactate**	**Total generated Lactate**
**4T1**	97% ± 0.18%	0.12% ± 0.021%	0.32% ± 0.025%	0.033% ± 0.0067%	0.20% ± 0.017%	2.2% ± 0.16%	26 ± 2.4 (μmol/million cell/12 h)
**Hela**	97% ± 0.24%	0.13% ± 0.050%	0.34% ± 0.071%	0.032% ± 0.0092%	0.29% ± 0.048%	2.3% ± 0.093%	40 ± 3.5 (μmol/million cell/12 h)
**K562**	98% ± 0.21%	0.11% ± 0.032%	0.28% ± 0.036%	0.016% ± 0.0079%	0.29% ± 0.029%	1.5% ± 0.12%	7.9 ± 0.90 (μmol/million cell/12 h)
**Thymocyte**	90% ± 0.17%	0.23% ± 0.022%	0.41% ± 0.031%	0.36% ± 0.030%	0.80% ± 0.041%	7.9% ± 0.19%	62 ± 0.83 (nmol/million cell/12 h)

4T1, Hela, K562 and thymocyte were incubated in serum-free RPMI 1640 medium containing 6 mM [U-^13^C]glucose for 12 hours in a humidified CO_2_ incubator and culture supernatant was collected for LC-MS/MS analysis as described in Materials and Methods. Data are mean ± SD, n = 12, from 2 independent experiments.

### Determination of lactate from glycolysis and pentose phosphate pathway

[1,2-^13^C_2_]glucose and [3-^13^C]glucose could be used for tracing lactate from glycolysis and from pentose phosphate pathway then back to glycolysis.

When [1,2-^13^C_2_]glucose was used to trace lactate in 4T1 cells, various lactate isotopomers and isotopologues were identified (the upper part of Table 2). Then we calculated the percentage of lactate from glycolysis, through pentose phosphate pathway then back to glycolysis according to the following estimation:

1. In theory, glycolysis would generate 2 species, [2,3-^13^C_2_]lactate (*m*/*z* 91 > 45) and lactate (*m*/*z* 89 > 43), which were derived from glucose carbons 1, 2, 3 and glucose carbon 4, 5, 6, respectively (Fig. 3);

**Figure 3:**
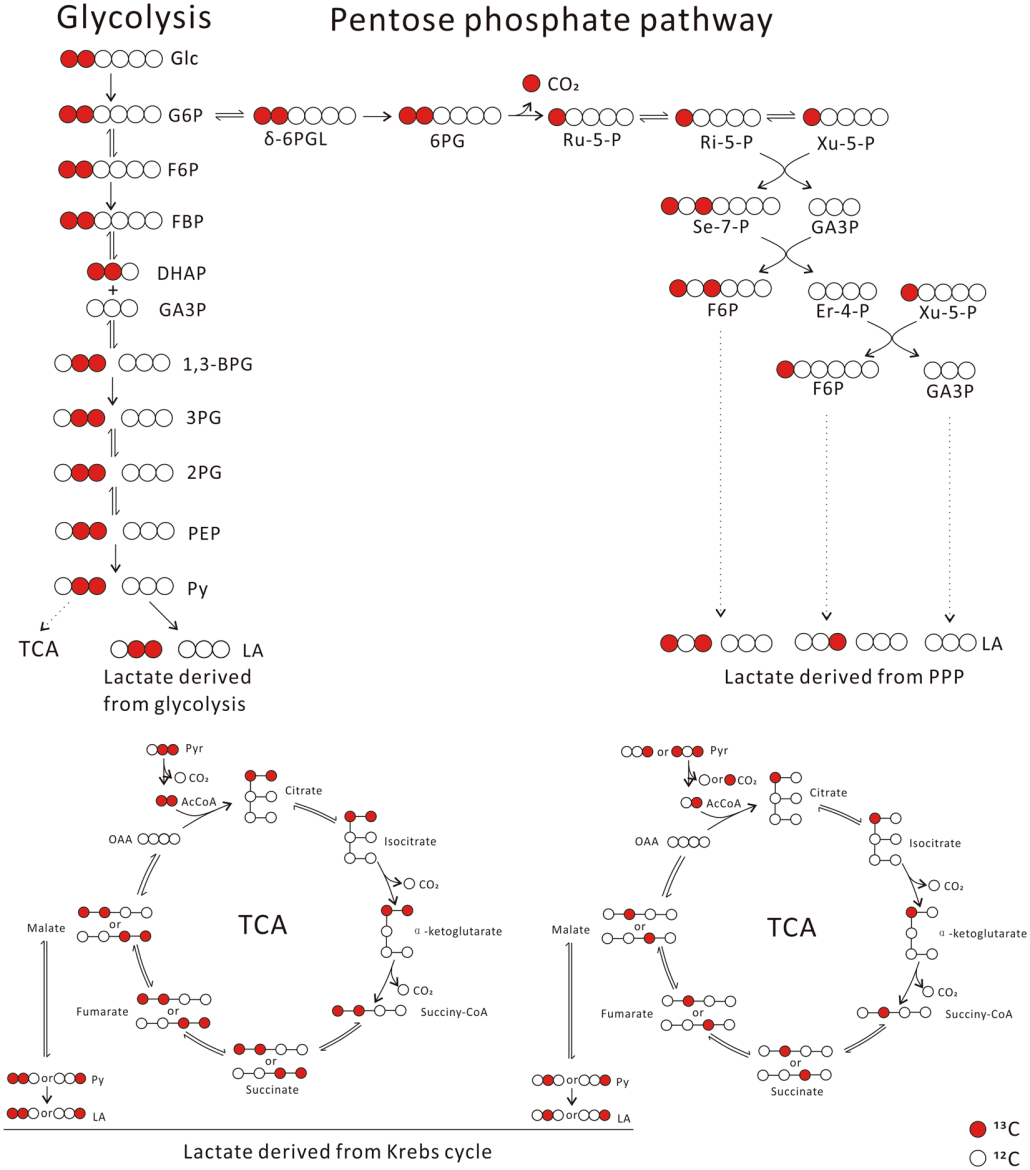
Metabolic pathways that generate various lactate isotopomers and isotopologues in cells, traced with [1,2-^13^C_2_]glucose. 4T1 cells were incubated in serum-free RPMI 1640 medium containing 6 mM [1,2-^13^C_2_]glucose for 12 hours in a humidified CO_2_ incubator and culture supernatant was collected for LC-MS analysis as described in Materials and Methods. The figure explain the generation of various lactate isotopologues from [1,2-^13^C_2_]glucose.
2. Glucose metabolism through pentose phosphate pathway then back to glycolysis would generated 3 major species (Fig. 3), lactate (*m*/*z* 89 > 43), [1,3-^13^C_2_]lactate (*m*/*z* 91 > 44), and [3-^13^C]lactate (*m*/*z* 90 > 44), the ratio of ([1,3-^13^C_2_]lactate + [3-^13^C]lactate)/lactate should be 2/3 (Fig. 3). Thus, glucose entering into pentose phosphate pathway then back to glycolysis would also generate a fraction of lactate (*m*/*z* 89 > 43).
3. According to Table 1, the other sources generated about 2% of lactate (*m*/*z* 89 > 43).

**Table 2:** The percentage of lactate isotopomers and isotopologues (mean ± SD, n = 12) generated by 4T1, Hela, K562, and thymocytes, traced by [1,2-^13^C_2_]glucose (upper part), the percentage of lactate (mean ± SD, n = 12) derived from glycolysis, PPP and other sources (down part).

	[2,3-^13^C_2_]lactate	lactate	[2-^13^C]- or [3-^13^C]lactate	[1,2-^13^C_2_]- or [1,3-^13^C_2_]lactate	[1-^13^C]lactate	[U-^13^C]lactate	Total generated Lactate
**4T1**	56% ± 0.43%	40% ± 0.36%	1.9% ± 0.099%	0.74% ± 0.039%	0.043% ± 0.032%	1.1% ± 0.041%	27 ± 2.4 (μmol/million cell/12 h)
**Hela**	55% ± 1.1%	41% ± 0.55%	2.4% ± 0.25%	0.81% ± 0.13%	0.11% ± 0.10%	1.5% ± 0.18%	40 ± 1.3 (μmol/million cell/12 h)
**K562**	56% ± 0.41%	40% ± 0.46%	2.1% ± 0.069%	0.83% ± 0.046%	0.054% ± 0.032%	1.1% ± 0.062%	7.7 ± 0.90 (μmol/million cell/12 h)
**Thymocytes**	52% ± 0.48%	44% ± 0.40%	1.8% ± 0.15%	0.59% ± 0.037%	0.19% ± 0.052%	1.3% ± 0.021%	60 ± 1.7 (nmol/million cell/12 h)
	**Lactate derived from glycolysis**		**Lactate derived from nonoxidative PPP**			**Lactate derived from other sources**	
	**Glc carbon 1,2,3**	**Glc carbon 4,5,6**	**Glc carbon 2,3**		**Glc carbon 4,5,6**		
	[2,3-^13^C_2_]lactate	lactate	[3-^13^C]lactate	[1,3-^13^C_2_]lactate	lactate	lactate	
**4T1**	56% ± 0.43%	34% ± 0.41%	1.9% ± 0.099%	0.74% ± 0.039%	4.0% ± 0.18%	2.2% ± 0.16%	
**sum**	90%		6.6%				
**Hela**	55% ± 1.1%	33% ± 0.26%	2.4% ± 0.25%	0.81% ± 0.13%	4.7% ± 0.57%	2.3% ± 0.093%	
**sum**	88%		7.8%				
**K562**	56% ± 0.41%	34% ± 0.64%	2.1% ± 0.069%	0.83% ± 0.046%	4.4% ± 0.17%	1.5% ± 0.12%	
**sum**	90%		7.3%				
**Thymocytes**	52% ± 0.48%	33% ± 0.48%	1.8% ± 0.15%	0.59% ± 0.037%	3.6% ± 0.25%	7.9% ± 0.19%	
**sum**	85%		6.0%				

4T1, Hela, K562 and thymocyte were incubated in serum-free RPMI-1640 medium containing 6 mM [1,2-^13^C_2_]glucose for 12 hours in a humidified CO_2_ incubator and culture supernatant was collected for LC-MS/MS analysis as described in Materials and Methods. The upper part summarizes the percentage of all lactate isotopologues. The down part assigned the percentage of isotopologues that are generated from glycolysis, pentose phosphate pathway (calculated based on percentage of [3-^13^C]- and [1,3-^13^C_2_]lactate as described in corresponding text), and other sources (according to percentage listed in Table 1). Data are mean ± SD, n = 12, from 2 independent experiments.

Thus, the percentage of lactate from glycolysis, pentose phosphate pathway, and other sources were about 90%, 6.6%, and 2.2%, respectively (the down part of Table 2), accounting for about 99% of total lactate. Using Hela and K562, we obtained nearly identical results (Table 2). For thymocytes, the percentage of lactate from glycolysis, pentose phosphate pathway, and other sources were about 85%, 6.0%, and 7.9%, respectively, accounting for about 99% of total lactate (Table 2).

**Figure 4:**
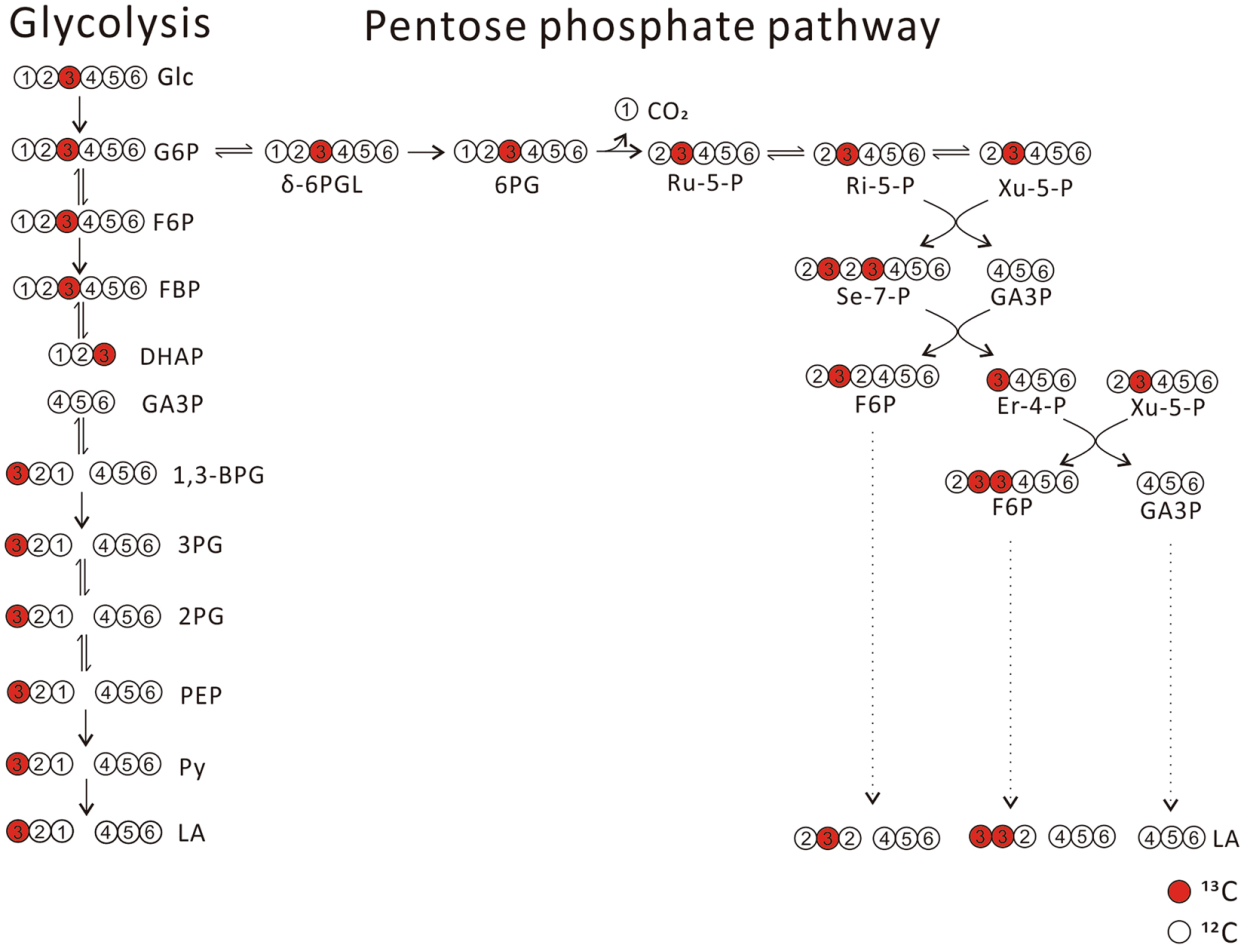
Metabolic pathways that generate various lactate isotopomers and isotopologues in cells, traced with [3-^13^C]glucose. In theory, when cells incubated with [3-^13^C]glucose, most glucose is converted to lactate directly through glycolysis, which results in equal amount of [1-^13^C]lactate that derived from glucose carbon 1, 2, 3 to lactate derived from glucose carbon 4, 5, 6. A fraction of glucose enters oxidative branch pentose phosphate pathway, cycles back to glycolysis through nonoxidative branch of pentose phosphate pathway, subsequently converts to lactate. Three major species, [1,2-^13^C_2_]lactate, [2-^13^C]lactate, and lactate, were generated from nonoxidative pentose phosphate pathway.

Then we used [3-^13^C]glucose to trace lactate generation from glycolysis and pentose phosphate pathway by 4T1 cells and calculated the percentage of lactate (Table 3). The calculation is similar as described above. In theory (Fig. 4), glycolysis would generate 2 species, [1-^13^C]lactate (*m*/*z* 90 > 44) derived from glucose carbons 1, 2, 3, and, lactate (*m*/*z* 89 > 43) derived from glucose carbons 4, 5, 6; glucose metabolism through pentose phosphate pathway then back to glycolysis would generate 3 major species (Fig. 4), lactate (*m*/*z* 89 > 43), [1,2-^13^C_2_]lactate (*m*/*z* 91 > 44), and [2-^13^C]lactate (*m*/*z* 90 > 44), the ratio of ([1,2-^13^C_2_]lactate + [2-^13^C]lactate)/lactate should be 2/3. Thus, the percentage of lactate from glycolysis, pentose phosphate pathway, and other sources were about 89%, 9.2%, and 2.2%, respectively (Table 3).

**Table 3:** The percentage of lactate isotopomers and isotopologues (mean ± SD, n = 12) generated by 4T1, Hela, K562, and thymocytes, traced by [3-^13^C]glucose (upper part), the percentage of lactate (mean ± SD, n = 12) derived from glycolysis, PPP and other sources (down part).

	[1-^13^C]lactate	lactate	[1,2-^13^C_2_]- or [1,3-^13^C_2_]lactate	[2-^13^C]- or [3-^13^C]lactate	[U-^13^C]lactate	[2,3-^13^C_2_]lactate	Total generated Lactate
4T1	45% ± 0.30%	51% ± 0.33%	2.7% ± 0.088%	1.0% ± 0.075%	0.29% ± 0.042%	0.13% ± 0.024%	25 ± 2.5 (μmol/million cell/12 h)
Hela	43% ± 0.77%	52% ± 0.45%	3.2% ± 0.22%	1.1% ± 0.11%	0.40% ± 0.057%	0.15% ± 0.020%	38 ± 3.3 (μmol/million cell/12 h)
K562	44% ± 0.47%	51% ± 0.45%	2.8% ± 0.079%	1.2% ± 0.062%	0.32% ± 0.058%	0.15% ± 0.027%	7.8 ± 0.35 (μmol/million cell/12 h)
Thymocytes	41% ± 0.21%	56% ± 0.24%	2.2% ± 0.043%	1.1% ± 0.062%	0.36% ± 0.044%	0.21% ± 0.013%	62 ± 1.8 (nmol/million cell/12 h)
	**Lactate derived from glycolysis**		**Lactate derived from nonoxidative PPP**			**Lactate derived from other sources**	
	**Glc carbon 1,2,3**	**Glc carbon 4,5,6**	**Glc carbon 2,3**		**Glc carbon 4,5,6**		
	[1-^13^C]lactate	lactate	[1,2-^13^C_2_]lactate	[2-^13^C]lactate	lactate	lactate	
4T1	45% ± 0.30%	44% ± 0.49%	2.7% ± 0.088%	1.0% ± 0.075%	5.5% ± 0.20%	2.2% ± 0.16%	
sum	89%		9.2%				
Hela	43% ± 0.77%	44% ± 0.37%	3.2% ± 0.22%	1.1% ± 0.11%	6.5% ± 0.48%	2.3% ± 0.093%	
sum	87%		11%				
K562	44% ± 0.47%	44% ± 0.49%	2.8% ± 0.079%	1.2% ± 0.062%	5.9% ± 0.19%	1.5% ± 0.12%	
sum	88%		9.9%				
Thymocytes	41% ± 0.21%	43% ± 0.37%	2.2% ± 0.043%	1.1% ± 0.062%	4.9% ± 0.15%	7.9% ± 0.19%	
sum	84%		8.1%				

4T1, Hela, K562 and thymocyte were incubated in serum-free RPMI-1640 medium containing 6 mM [3-^13^C]glucose for 12 hours in a humidified CO_2_ incubator and culture supernatant was collected for LC-MS/MS analysis as described in Materials and Methods. The upper part summarizes the percentage of each lactate isotopologues. The down part assigned the percentage of isotopologues that are generated from glycolysis, pentose phosphate pathway, and other sources. Specifically, the percentage of lactate is contributed by three parts, from glycolysis, pentose phosphate pathway (calculated based on percentage of [2-^13^C]- and [1,2-^13^C_2_]lactate as described in corresponding text), and other sources (according to percentage listed in Table 1). Data are mean ± SD, n = 12, from 2 independent experiments.

Using Hela and K562, we obtained similar results (Table 3). For thymocytes, the percentage of lactate from glycolysis, pentose phosphate pathway, and other sources were about 84%, 8.1%, and 7.9%, respectively (Table 3).

### The quantification problem of lactate generated from glycolysis

When [1,2-^13^C_2_]glucose was used for tracing, [2,3-^13^C_2_]lactate (*m*/*z* 91 > 45, derived from glucose carbons 1, 2, 3) and lactate (*m*/*z* 89 > 43, derived from glucose carbons 4, 5, 6) from glycolysis constituted 56% ± 0.43% and 34% ± 0.41% of total lactate, respectively, resulting in a ratio of 1.6 (Table 2). This is a surprise, as this ratio should be 1.

When using [3-^13^C]glucose for tracing, [1-^13^C]lactate (*m*/*z* 90 > 43, derived from glucose carbons 1, 2, 3) and lactate (*m*/*z* 89 > 43, derived from glucose carbons 4, 5, 6) from glycolysis were 45% ± 0.30% and 44% ± 0.49% of total lactate, respectively, resulting in a ratio of 1.02, very close to 1 (Table 3).

Using 4T1, Hela, k562, and mouse thymocytes, we obtained nearly identical results (Tables 2–3), indicating that the results were highly reproducible and reliable.

### Tracing lactate isotopomers and isotopologues using glucose labeled with carbon-13 at different positions

We then used [1-^13^C]glucose, [6-^13^C]glucose, [1,2,3-^13^C_3_]glucose, and [4-^13^C]glucose to trace lactate. The percentages of each lactate isotopologues in 4T1 were given in the upper part of Table 4. Then we estimated the percentage of lactate generated from glycolysis, pentose phosphate pathway, and other sources (the down part of Table 4).

**Table 4:** The percentage of lactate isotopomers and isotopologues (mean ± SD, n = 12) generated by 4T1, traced by [1-^13^C]-, [6-^13^C]-, [1,2,3-^13^C_3_]-, and [4-^13^C]glucose (upper part), the percentage of lactate (mean ± SD, n = 12) derived from glycolysis, PPP and other sources (down part).

^13^C-labeled glucose	[2-^13^C]- [3-^13^C]lactate	lactate	[1,2-^13^C_2_]- or [1,3-^13^C_2_]lactate	[1-^13^C]lactate	[U-^13^C]lactate	[2,3-^13^C_2_]lactate	Total generated Lactate
**[1-** ^**13**^ **C]glucose**	49% ± 0.45%	49% ± 0.45%	0.79% ± 0.030%	0.14% ± 0.036%	0.024% ± 0.0081%	0.96% ± 0.084%	26 ± 2.4 (μmol/million cell/12 h)
**[6-** ^**13**^ **C]glucose**	46% ± 0.46%	52% ± 0.50%	0.046% ± 0.018%	0.024% ± 0.0037%	0.96% ± 0.066%	0.71% ± 0.039%	26 ± 1.6 (μmol/million cell/12 h)
**[1,2,3-** ^**13**^ **C**_**3**_ **]glucose**	57% ± 0.38%	42% ± 0.36%	0.30% ± 0.039%	0.16% ± 0.018%	0.043% ± 0.030%	0.22% ± 0.022%	26 ± 1.2 (μmol/million cell/12 h)
**[4-** ^**13**^ **C]glucose**	50% ± 0.34%	48% ± 0.33%	0.41% ± 0.054%	1.5% ± 0.054%	0.15% ± 0.014%	0.073% ± 0.013%	25 ± 1.1 (μmol/million cell/12 h)
^**13**^ **C-labeled glucose**	**Lactate derived from glycolysis**		**Lactate derived from nonoxidative PPP**		**Lactate derived from other sources**		
	**Glc carbon 1,2,3**	**Glc carbon 4,5,6**	**Glc carbon 2,3**	**Glc carbon 4,5,6**	**lactate**		
**[1-** ^**13**^ **C]glucose**	49% ± 0.45%	37% ± 0.36%	3.7% ± 0.0%	5.5% ± 0.0%	2.2% ± 0.16%		
**sum**	86%		9.2%				
**[6-** ^**13**^ **C]glucose**	40% ± 0.55%	47% ± 0.50%	3.7% ± 0.00%	5.5% ± 0.00%	2.2% ± 0.16%		
**sum**	87%		9.2%				
**[1,2,3-** ^**13**^ **C**_**3**_ **]glucose**	54% ± 0.38%	34% ± 0.33%	3.7% ± 0.00%	5.5% ± 0.00%	2.2% ± 0.16%		
**sum**	88%		9.2%				
**[4-** ^**13**^ **C]glucose**	44% ± 0.40%	43% ± 0.33%	3.7% ± 0.0%	5.5% ± 0.0%	2.2% ± 0.16%		
**sum**	87%		9.2%				

4T1 cells were incubated in serum-free RPMI-1640 medium containing 6 mM [1-^13^C]glucose (or [6-^13^C]-, [1,2,3-^13^C_3_]-, [4-^13^C]glucose) for 12 hours in a humidified CO_2_ incubator and culture supernatant was collected for LC-MS/MS analysis as described in Materials and Methods. The upper part summarizes the percentage of all lactate isotopologues. The down part assigned the percentage of isotopologues that are generated from glycolysis, pentose phosphate pathway, and other sources. Specifically, the percentage of lactate is composed of three parts, from glycolysis, pentose phosphate pathway (calculated based on percentage of [2-^13^C]lactate and [1,2-^13^C_2_]lactate in Table 3), and other sources (according to percentage listed in Table 1). Data are mean ± SD, n = 12, from 2 independent experiments.

Again, notably, the ratio of lactate derived from glucose carbon 1, 2, and 3 over that from glucose carbon 4, 5, and 6 via glycolysis varied significantly in 4T1 cells, ranging from 1.6 ([1,2,3-^13^C_3_]glucose) to 0.85 ([6-^13^C]glucose) (the down part of Table 4). These results were reproducible using Hela, K562, and mouse thymocytes (Supplementary Tables S7–S10).

### ^13^C-labelling of lactate at different positions differentially influences its fragmentation efficiency under tandem mass spectrometry condition

In theory, glucose labeling with ^13^C should not alter the percentage ratio between lactate derived from glucose carbon 1, 2, 3 and lactate derived from glucose carbon 4, 5, 6, because there is no theoretical and experimental basis that enzymes responsible for glucose metabolism can distinguish ^13^C from ^12^C.

According to the methodology of mass spectrometry, isotopomers or isotopologues which differ only at ^13^C or ^12^C should not affect the ionization and fragmentation, so that the percentage of each isotopomer or isotopologue of a metabolite could be quantified. In fact, this is the principle used to measure isotopomers or isotopologues of metabolites in metabolomic studies^28–31^.

However, after generating so many confusing data as described above, we suspected that carbon-13 may significantly interfere with molecule fragmentation under tandem mass spectrometry condition and interfere the quantification of each lactate isotopomer and isotopologue.

**Figure 5:**
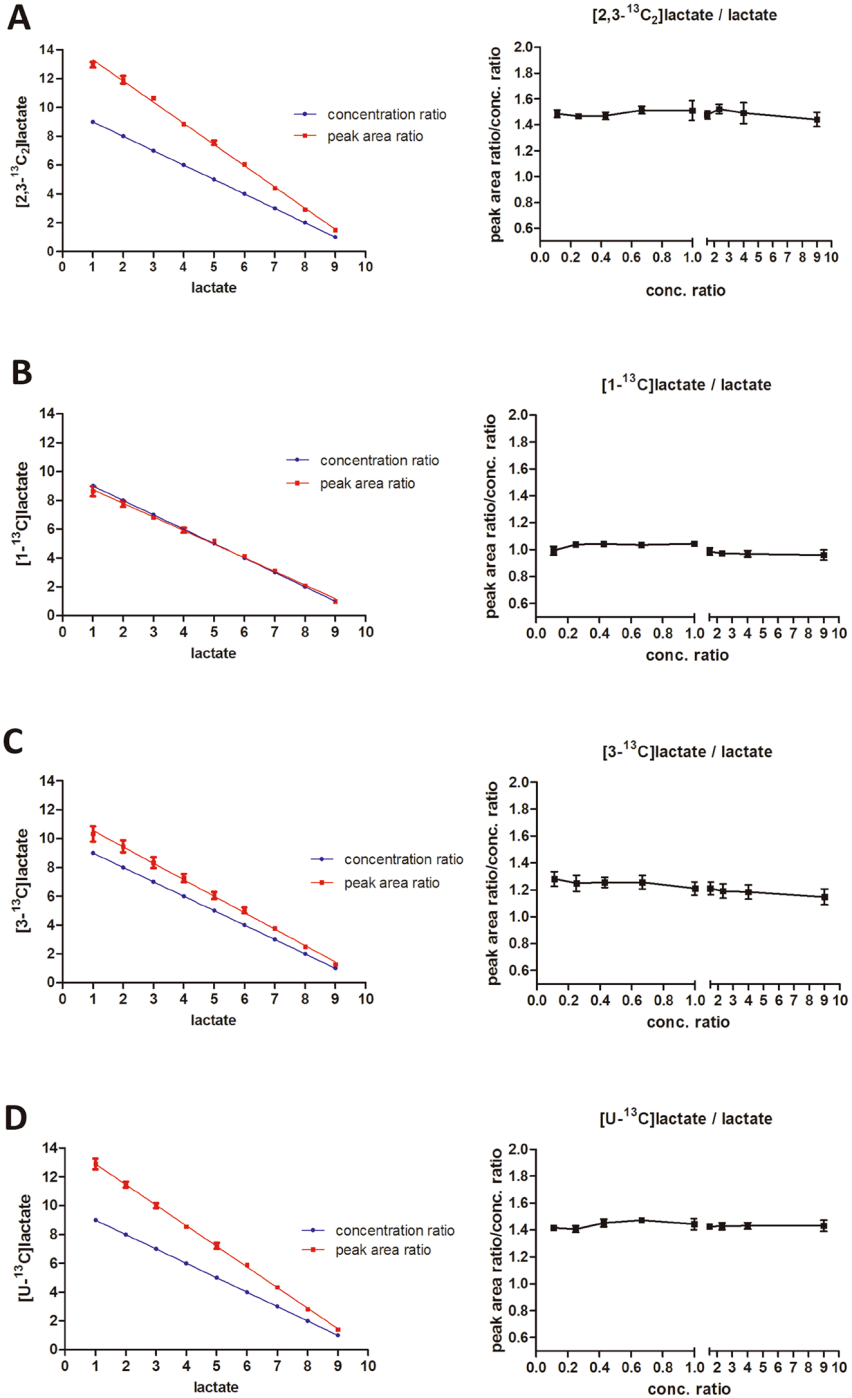
^13^C-labeling of lactate at different positions differentially influences its fragmentation efficiency under tandem mass spectrometry condition. (**A**) Left panel: standard mixture ([2,3-^13^C_2_]lactate: lactate); red line: peak area ratios; blue line: concentration ratios. The mixtures were analyzed by LC-MS/MS under MRM mode in losing CO. [2,3-^13^C_2_]lactate/lactate peak area ratio was 1.5 folds higher than [2,3-^13^C_2_]lactate/lactate concentration ratio (right panel). Original data refers to Supplementary Table 11. (**B**) Left panel: standard mixture ([1-^13^C]lactate: lactate); red line: peak area ratios; blue line: concentration ratios. The mixtures were analyzed by LC-MS/MS under MRM mode in losing CO. [1-^13^C]lactate/lactate peak area ratio was comparable with [1-^13^C]lactate/lactate concentration ratio (right panel). Original data refers to Supplementary Table 12. (**C**) Left panel: standard mixture ([3-^13^C]lactate: lactate); red line: peak area ratios; blue line: concentration ratios. The mixtures were analyzed by LC-MS/MS under MRM mode in losing CO, [3-^13^C]lactate/lactate peak area ratio was 1.2 folds higher than [3-^13^C]lactate/lactate concentration ratio (right panel). Original data refers to Supplementary Table 13. (**D**) Left panel: standard mixture ([U-^13^C]lactate: lactate); red line: peak area ratios; blue line: concentration ratios. The mixtures were analyzed by LC-MS/MS under MRM mode in losing CO, [U-^13^C]lactate/lactate peak area ratio was 1.4 folds higher than [U-^13^C]lactate/lactate concentration ratio (right panel). Original data refers to Supplementary Table 14. The data represent mean ± SD, n = 6, and are confirmed by 2 independent experiments.

Standards [2,3-^13^C_2_]lactate was mixed with lactate, and the [2,3-^13^C_2_]lactate/lactate concentration ratios were: 9 (9:1); 4 (8:2); 2.3 (7:3); 1.5 (6:4); 1.0 (5:5); 0.67 (4:6); 0.43 (3:7); 0.25 (2:8) and 0.11 (1:9). The mixtures were analyzed by LC-MS/MS under MRM mode in losing CO, and results shown that [2,3-^13^C_2_]lactate/lactate peak area ratio were 13 ± 0.45; 6.0 ± 0.29; 3.6 ± 0.077; 2.2 ± 0.042; 1.5 ± 0.070; 1.0 ± 0.018; 0.63 ± 0.011; 0.37 ± 0.0037 and 0.17 ± 0.0029, respectively (Supplementary Table S11). Overall, [2,3-^13^C_2_]lactate/lactate peak area ratio was 1.5 folds higher than [2,3-^13^C_2_]lactate/lactate concentration ratio (the right panel of Fig. 5A), indicating that ^13^C enhanced the fragmentation rate of [2,3-^13^C_2_]lactate under MRM mode in losing CO.

Then we mixed [1-^13^C]-, [3-^13^C]-, or [U-^13^C]lactate with lactate respectively, and their concentration ratios, analysis and calculation methods were similar as described above. While [1-^13^C]lactate/lactate peak area ratio was comparable with their concentration ratio (the right panel of Fig. 5B), [3-^13^C]lactate/lactate and [U-^13^C]lactate/lactate peak area ratio was 1.2 (the right panel of Fig. 5C) and 1.4 (the right panel of Fig. 5D) folds higher than their concentration ratio, respectively, indicating that ^13^C-labelling of lactate at different positions differentially influences its fragmentation efficiency under tandem mass spectrometry condition.

The lactate and [2,3-^13^C_2_]lactate (or [1-^13^C]-, [3-^13^C]-, [U-^13^C]lactate) mixed standard solutions mentioned above were analyzed by LC-MS under Q1 multiple ions mode, in order to investigate whether ^13^C affects ionization process of ^13^C-labeled lactate. Data in Supplementary Tables S11–S14 point out that ^13^C slightly affect ionization of ^13^C-labeled lactate under MS condition.

In order to investigate ^13^C effect on lactate fragmentation under different MRM mode, the lactate and [2,3-^13^C_2_]lactate (or [1-^13^C]-, [3-^13^C]-, [U-^13^C]lactate) mixed standard solutions mentioned above were analyzed by MRM mode in losing H_2_O. The results pointed out that ^13^C enhanced fragmentation of [2,3-^13^C_2_]-, [3-^13^C]-, or [U-^13^C]lactate as well, but the influence was less than that under MRM mode in losing CO; and the fragmentation of [1-^13^C]lactate was not affected by ^13^C under MRM mode in losing H_2_O (Supplementary Tables S11–S14).

The results raised a theoretical issue: ^13^C and ^12^C differs in only one number of neutron, the chemical bond was formed by sharing the outer electrons and the strength of the bond was hardly influenced by the neutron. In tandem mass spectrometry, collision gas such as nitrogen was used and the fragmentation occurs when the collision energy was high enough to break the chemical bond. Under the same collision energy, the intensity of the fragment ion from the ^12^C- and ^13^C-labeled lactate was thought to be identical. Even if there is some difference due to the mass variability, it will be very little. However, in the present study, significant difference was observed.

### Data calibration

We used the number 1.5 ([2,3-^13^C_2_]lactate versus lactate) derived from Fig. 5 to calibrate the data acquired from 4T1 incubated with [1,2-^13^C_2_]glucose (Table 2). After calibration, the ratio of lactate derived from glucose carbon 1, 2, 3 over that from glucose carbon 4, 5, 6 was nearly equal (Table 5).

**Table 5:** Percentage of lactate isotopomers and isotopologues (mean ± SD, n = 12) generated by 4T1, traced by [1,2-^13^C_2_]-, [1-^13^C]-, [6-^13^C]- and [1,2,3-^13^C_3_]glucose before and after calibration.

		Lactate derived from glycolysis		Ratio of lactate derived from glucose carbon 1,2,3 over that derived from glucose carbon 4,5,6
		Glc carbon 1,2,3	Glc carbon 4,5,6	
**[1**,**2-** ^**13**^ **C**_**2**_ **]glucose**	**Before calibration**	56% ± 0.43%	34% ± 0.41%	1.6
	**After calibration**	47% ± 0.47%	42% ± 0.80%	1.1
**[1-** ^**13**^ **C]glucose**	**Before calibration**	49% ± 0.45%	37% ± 0.36%	1.3
	**After calibration**	45% ± 0.44%	41% ± 0.36%	1.1
**[6-** ^**13**^ **C]glucose**	**Before calibration**	40% ± 0.55%	47% ± 0.50%	0.85
	**After calibration**	44% ± 0.56%	43% ± 0.50%	1.0
**[1**,**2**,**3-** ^**13**^ **C**_**3**_ **]glucose**	**Before calibration**	54% ± 0.38%	34% ± 0.33%	1.6
	**After calibration**	46% ± 0.38%	41% ± 0.36%	1.1

Percentage of lactate isotopomers and isotopologues derived from [1,2-^13^C_2_]glucose is calibrated using 1.5 ([2,3-^13^C_2_]lactate VS lactate, the right panel of Fig. 5A). Percentage of ^13^C-labeling lactate derived from [1-^13^C]glucose and [6-^13^C]glucose is calibrated using 1.2 ([3-^13^C]lactate VS lactate, the right panel of Fig. 5C). Percentage of ^13^C-labeling lactate derived from [1,2,3-^13^C_3_]glucose is calibrated using 1.4 ([U-^13^C]lactate VS lactate, the right panel of Fig. 5D). The data before calibration are from Tables 2 and 4.

We used the number 1.2 ([3-^13^C]lactate versus lactate) derived from Fig. 5 to calibrate the data of 4T1 incubated with [1-^13^C]- or [6-^13^C]glucose (Table 4), the ratio of lactate derived from glucose carbon 1, 2, 3 over that from glucose carbon 4, 5, 6, was close to 1 (Table 5).

We used the number 1.4 ([U-^13^C]lactate versus lactate) derived from Fig. 5 to calibrate the data of 4T1 incubated with [1,2,3-^13^C_3_]glucose (Table 4). After calibration, the ratio of lactate derived from glucose carbon 1, 2, 3 over that from glucose carbon 4, 5, 6 was comparable with each other (Table 5).

Calibrations were performed on the data of Hela, K562, and thymocytes, the calibrated data indicated that the percentages of lactate derived from glucose carbon 1, 2, 3 and 4, 5, 6 were nearly equal as well (Supplementary Tables S15–S18).

### Theoretical calculation of the activation energy of lactate fragmentation

**Figure 6:**
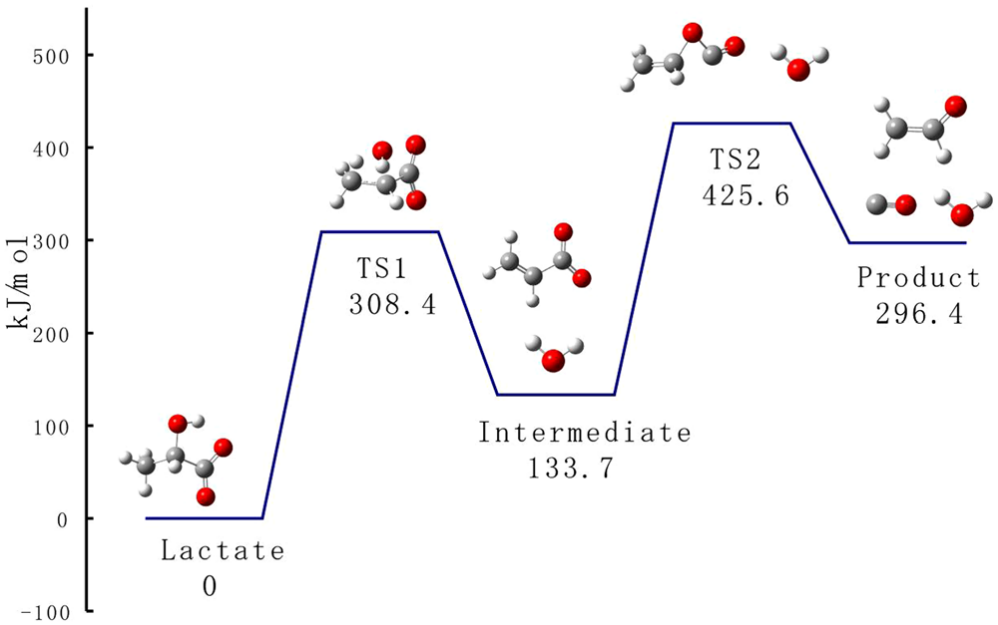
Potential energy diagram for the fragmentation reactions of lactate, using DFT calculations at the B3LYP/6-31 G(d) level.

To obtain insights into the mechanism of lactate fragmentation, we carried out theoretical calculations at the B3LYP/6-31 G(d) level of theory to quantitatively describe the energy requirements of these reactions^32–34^ and a schematic potential energy surface is illustrated in Fig. 6. The energy of transition state 2 (TS2) for losing CO is higher than that of transition state 1 (TS1) for losing H_2_O, suggesting that the loss of CO process is the rate determining step for the formation of ion at *m*/*z* 43., the relative energies of lactate isotopomers and isotopologues in the fragmentation reaction routes were shown in Table 6. The energies of TS2 of all these compounds were nearly identical, suggesting that lactate isotopomers and isotopologues should have identical fragmentation behaviors in tandem mass spectrometry. The calculation was inconsistent with the experimental data and this inconsistency is worthy of further investigation.

**Table 6:** Relative energies of species involved in the fragmentation reaction routes of lactate isotopomers and isotopologues.

Compound	Reactant (kJ/mol)	TS1 (kJ/mol)	Intermediate (kJ/mol)	TS2 (kJ/mol)	Product (kJ/mol)
lactate	0	308.4	133.7	425.6	296.4
[1-^13^C]lactate	0	308.4	133.7	425.8	296.7
[2-^13^C]lactate	0	308.5	133.7	425.7	296.4
[3-^13^C]lactate	0	308.4	133.7	425.6	296.4
[1,2-^13^C_2_]lactate	0	308.5	133.8	425.9	296.7
[1,3-^13^C_2_]lactate	0	308.4	133.7	425.9	296.8
[2,3-^13^C_2_]lactate	0	308.5	133.8	425.7	296.4
[U-^13^C]lactate	0	308.5	133.8	426.0	296.8

### Concluding remarks

To the best of our knowledge, there is no report regarding the significant impact of carbon-13 labeling on fragmentation of molecules under tandem MS condition. In this study, we revealed that carbon-13 labeling could significantly interfere with lactate fragmentation. This observation is inconsistent with our theoretical calculation that the activation energy of fragmentation of lactate with or without carbon-13 labeling is nearly identical. Our study also points out that potential problems may exist in the previous studies involving quantification of isotopomers and isotopologues by tandem mass spectrometry technology. In the future, the proper calibrations for quantification of isotopomers and isotopologues would be required.

## Electronic supplementary material

Supplementary information
