# Supplementary material for: Quantification of lactate from various metabolic pathways and quantification issues of lactate isotopologues and isotopmers
Source: Sci Rep. 2017 Aug 16;7:8489. doi: 10.1038/s41598-017-08277-3 (PMC5559627; doi:10.1038/s41598-017-08277-3)
Supplement: Supplementary file 1 — Supplementary information [file 41598_2017_8277_MOESM1_ESM.doc]

**Quantification of lactate from various metabolic pathways and quantification issues of lactate isotopologues and isotopmers**

**Wei Zhang^1#^, Cheng Guo^1#^, Kezhi Jiang^2^, Minfeng Ying^1^, Xun Hu^1^***

^1^ Cancer Institute (a Key Laboratory For Cancer Prevention & Intervention, China National Ministry of Education), The Second Affiliated Hospital, Zhejiang University School of Medicine, 88 Jiefang Road, Hangzhou, China.

^2^ Key Laboratory of Organosilicon Chemistry and Material Technology, Hangzhou Normal University, Hangzhou, China.

^#^ Equal contributors to the work

* Address correspondence to: Xun Hu, Ph.D., 88 Jiefang Road, Cancer Institute, Zhejiang University School of Medicine, Hangzhou, China, phone:86-571-87783656, Email: [huxun@zju.edu.cn](mailto:huxun@zju.edu.cn)

**Supplementary figures**

**A**


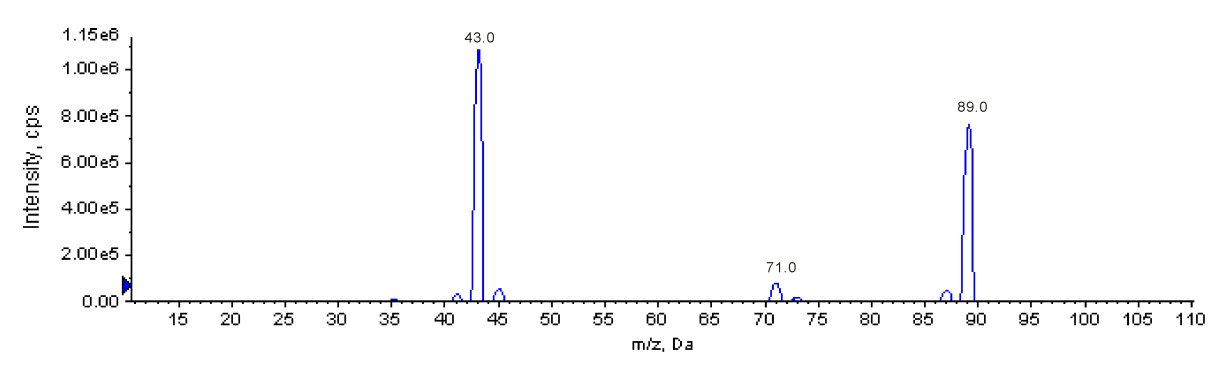


**B**


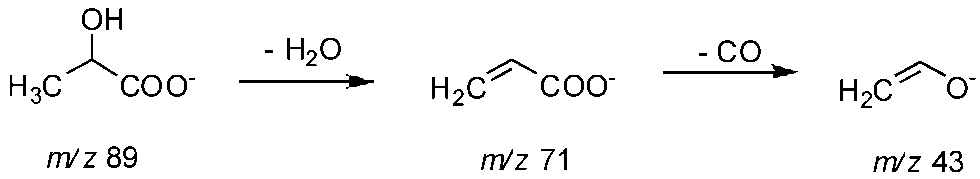


**Figure S1. A: The collision-induced dissociation spectrum of lactate, B: Proposed fragmentation mechanism of lactate**


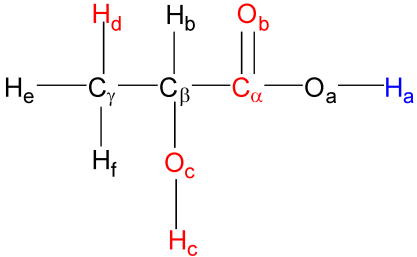


**Figure S2. Chemical structure of lactate.** The H in blue was lost during electrospray ionization; the C, H and O in red were lost in MS/MS dissociation.

Q1

Q3

Q2

detector

Ion source

isolation

isolation

dissociation

**Figure S3. The rationale for quantification of metabolites under MRM mode of mass spectrometer with QTRAP analyzer**

**
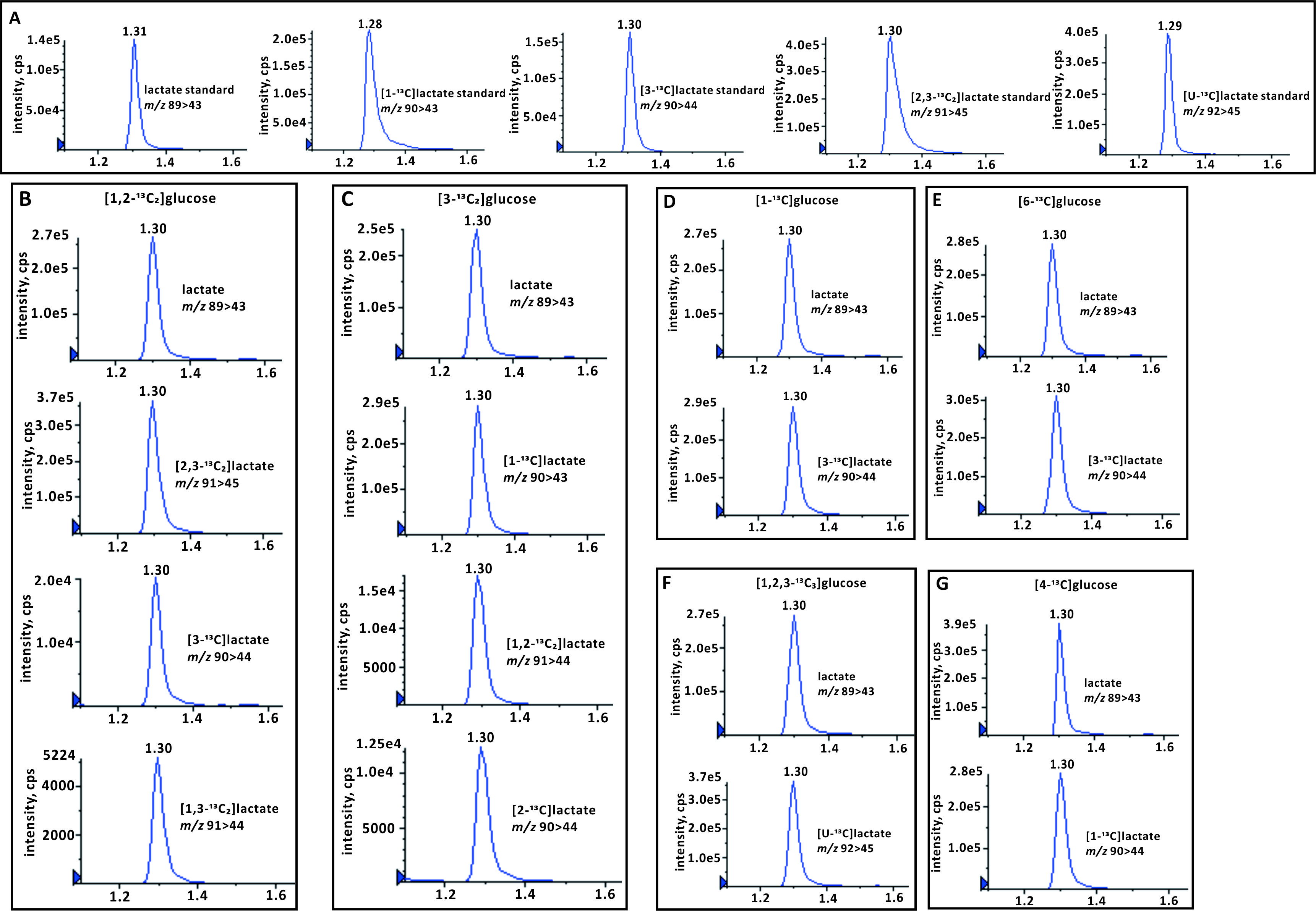
**

**Figure S4.** A: Representative ion chromatogram of lactate isotopomer and isotopologue standard. B: Selected ion chromatograms of four major lactate species generated by [1,2-^13^C_2_]glucose in 4T1: lactate, [2,3-^13^C_2_]-, [1,3-^13^C_2_]-, and [3-^13^C]lactate, the sum of which was 99% of total generated lactate. C: Selected ion chromatograms of four major lactate species generated by [3-^13^C]glucose in 4T1: lactate, [1-^13^C]-, [1,2-^13^C_2_]-, and [2-^13^C]lactate, the sum of which was more than 99% of total generated lactate. D: Representative ion chromatograph of two major lactate species generated by [1-^13^C]glucose in 4T1: lactate and [3-^13^C]lactate, the sum of which was 98% of total generated lactate. E: Representative ion chromatographs of two major lactate species generated by [6-^13^C]glucose in 4T1: lactate and [3-^13^C]lactate, the sum of which was 98% of total generated lactate. F: Representative ion chromatographs of two major lactate species generated by [1,2,3-^13^C_3_]glucose in 4T1: lactate and [U-^13^C]lactate, the sum of which was 99% of total generated lactate. G: Representative ion chromatographs of two major lactate species generated by [4-^13^C]glucose in 4T1: lactate and [1-^13^C]lactate, the sum of which was 98% of total generated lactate.

**Supplementary tables**

**Supplementary Table S1. The optimized MS conditions used for the analysis of the target analytes**

| **Compound** | **MRM**  **ion transition (*m/z*)** | **DP (V)** | **CE (V)** | **EP (V)** | **CXP (V)** |
| --- | --- | --- | --- | --- | --- |
| **lactate** | 89.0>43.0 | -60.0 | -22.0 | -10.0 | -5.0 |
|  | 89.0>71.0 | -40.0 | -15.0 | -5.0 | -5.0 |
|  | 89.0 | -40.0 | / | -10.0 | / |
| **[1-^13^C]lactate** | 90.0>43.0 | -60.0 | -22.0 | -10.0 | -5.0 |
|  | 90.0>72.0 | -40.0 | -15.0 | -5.0 | -5.0 |
|  | 90.0 | -40.0 | / | -10.0 | / |
| **[2-^13^C]lactate** | 90.0>44.0 | -60.0 | -22.0 | -10.0 | -5.0 |
|  | 90.0>72.0 | -40.0 | -15.0 | -5.0 | -5.0 |
|  | 90.0 | -40.0 | / | -10.0 | / |
| **[3-^13^C]lactate** | 90.0>44.0 | -60.0 | -22.0 | -10.0 | -5.0 |
|  | 90.0>72.0 | -40.0 | -15.0 | -5.0 | -5.0 |
|  | 90.0 | -40.0 | / | -10.0 | / |
| **[1,2-^13^C_2_]lactate** | 91.0>44.0 | -60.0 | -22.0 | -10.0 | -5.0 |
|  | 91.0>73.0 | -40.0 | -15.0 | -5.0 | -5.0 |
|  | 91.0 | -40.0 | / | -10.0 | / |
| **[1,3-^13^C_2_]lactate** | 91.0>44.0 | -60.0 | -22.0 | -10.0 | -5.0 |
|  | 91.0>73.0 | -40.0 | -15.0 | -5.0 | -5.0 |
|  | 91.0 | -40.0 | / | -10.0 | / |
| **[2,3-^13^C_2_]lactate** | 91.0>45.0 | -60.0 | -22.0 | -10.0 | -5.0 |
|  | 91.0>73.0 | -40.0 | -15.0 | -5.0 | -5.0 |
|  | 91.0 | -40.0 | / | -10.0 | / |
| **[U-^13^C]lactate** | 92.0>45.0 | -60.0 | -22.0 | -10.0 | -5.0 |
|  | 92.0>74.0 | -40.0 | -15.0 | -5.0 | -5.0 |
|  | 92.0 | -40.0 | / | -10.0 | / |

*DP* declustering potential, *CE* collision energy, *EP* entrance potential, *CXP* collision cell exit potential

**Table S2. Relative isotopic abundances of carbon, hydrogen and oxygen elements.**

| **C** | | **H** | | **O** | | |
| --- | --- | --- | --- | --- | --- | --- |
| ^12^C | ^13^C | ^1^H | ^2^H | ^16^O | ^17^O | ^18^O |
| 98.93% | 1.07% | 99.99% | 0.011% | 99.76% | 0.038% | 0.205% |

**Table S3. Contributions from natural isotopmers of lactate to the formation of ion transition *m/z*** **89>43, 90>43, 90>44, 91>44, 91>45, 92>45 (the C, H, O in black stands for ^12^C, ^1^H, ^16^O, respectively; the C, H, O in red stands for ^13^C, ^2^H, ^18^O, respectively; the O in blue stands for ^17^O).**

| **Natural isotopmer of lactate, C_3_H_5_O_3_^-^, that could produce transition *m/z* 89>43** | **The percentage of this natural isotopmer of lactate** |
| --- | --- |
| C_α_C_β_C_γ_H_b_H_c_H_d_H_e_H_f_O_a_O_b_O_c_ | 96.08071% |
| **Natural isotopmers of lactate, C_3_H_5_O_3_^-^, that could produce transition *m/z* 90>43** | **The percentage of these natural isotopmers of lactate** |
| C_α_C_β_C_γ_H_b_H_c_H_d_H_e_H_f_O_a_O_b_O_c_ | 1.03918% |
| C_α_C_β_C_γ_H_b_H_c_H_d_H_e_H_f_O_a_O_b_O_c_ | 0.01057% |
| C_α_C_β_C_γ_H_b_H_c_H_d_H_e_H_f_O_a_O_b_O_c_ | 0.01057% |
| C_α_C_β_C_γ_H_b_H_c_H_d_H_e_H_f_O_a_O_b_O_c_ | 0.03660% |
| C_α_C_β_C_γ_H_b_H_c_H_d_H_e_H_f_O_a_O_b_O_c_ | 0.03660% |
| sum | 1.13352% |
| **Natural isotopmers of lactate, C_3_H_5_O_3_^-^, that could produce transition *m/z* 90>44** | **The percentage of these natural isotopmers of lactate** |
| C_α_C_β_C_γ_H_b_H_c_H_d_H_e_H_f_O_a_O_b_O_c_ | 1.03918% |
| C_α_C_β_C_γ_H_b_H_c_H_d_H_e_H_f_O_a_O_b_O_c_ | 1.03918% |
| C_α_C_β_C_γ_H_b_H_c_H_d_H_e_H_f_O_a_O_b_O_c_ | 0.01057% |
| C_α_C_β_C_γ_H_b_H_c_H_d_H_e_H_f_O_a_O_b_O_c_ | 0.01057% |
| C_α_C_β_C_γ_H_b_H_c_H_d_H_e_H_f_O_a_O_b_O_c_ | 0.01057% |
| C_α_C_β_C_γ_H_b_H_c_H_d_H_e_H_f_O_a_O_b_O_c_ | 0.03660% |
| sum | 2.14667% |
| **Natural isotopmers of lactate, C_3_H_5_O_3_^-^, that could produce transition *m/z* 91>44** | **The percentage of these natural isotopmers of lactate** |
| C_α_C_β_C_γ_H_b_H_c_H_d_H_e_H_f_O_a_O_b_O_c_ | 0.01124% |
| C_α_C_β_C_γ_H_b_H_c_H_d_H_e_H_f_O_a_O_b_O_c_ | 0.01124% |
| C_α_C_β_C_γ_H_b_H_c_H_d_H_e_H_f_O_a_O_b_O_c_ | 0.00000% |
| C_α_C_β_C_γ_H_b_H_c_H_d_H_e_H_f_O_a_O_b_O_c_ | 0.00000% |
| C_α_C_β_C_γ_H_b_H_c_H_d_H_e_H_f_O_a_O_b_O_c_ | 0.00000% |
| C_α_C_β_C_γ_H_b_H_c_H_d_H_e_H_f_O_a_O_b_O_c_ | 0.00000% |
| C_α_C_β_C_γ_H_b_H_c_H_d_H_e_H_f_O_a_O_b_O_c_ | 0.00000% |
| C_α_C_β_C_γ_H_b_H_c_H_d_H_e_H_f_O_a_O_b_O_c_ | 0.00000% |
| C_α_C_β_C_γ_H_b_H_c_H_d_H_e_H_f_O_a_O_b_O_c_ | 0.00011% |
| C_α_C_β_C_γ_H_b_H_c_H_d_H_e_H_f_O_a_O_b_O_c_ | 0.00011% |
| C_α_C_β_C_γ_H_b_H_c_H_d_H_e_H_f_O_a_O_b_O_c_ | 0.00011% |
| C_α_C_β_C_γ_H_b_H_c_H_d_H_e_H_f_O_a_O_b_O_c_ | 0.00011% |
| C_α_C_β_C_γ_H_b_H_c_H_d_H_e_H_f_O_a_O_b_O_c_ | 0.00011% |
| C_α_C_β_C_γ_H_b_H_c_H_d_H_e_H_f_O_a_O_b_O_c_ | 0.00011% |
| C_α_C_β_C_γ_H_b_H_c_H_d_H_e_H_f_O_a_O_b_O_c_ | 0.00011% |
| C_α_C_β_C_γ_H_b_H_c_H_d_H_e_H_f_O_a_O_b_O_c_ | 0.00040% |
| C_α_C_β_C_γ_H_b_H_c_H_d_H_e_H_f_O_a_O_b_O_c_ | 0.00040% |
| C_α_C_β_C_γ_H_b_H_c_H_d_H_e_H_f_O_a_O_b_O_c_ | 0.00040% |
| C_α_C_β_C_γ_H_b_H_c_H_d_H_e_H_f_O_a_O_b_O_c_ | 0.00040% |
| C_α_C_β_C_γ_H_b_H_c_H_d_H_e_H_f_O_a_O_b_O_c_ | 0.00040% |
| C_α_C_β_C_γ_H_b_H_c_H_d_H_e_H_f_O_a_O_b_O_c_ | 0.00000% |
| C_α_C_β_C_γ_H_b_H_c_H_d_H_e_H_f_O_a_O_b_O_c_ | 0.00000% |
| C_α_C_β_C_γ_H_b_H_c_H_d_H_e_H_f_O_a_O_b_O_c_ | 0.00000% |
| C_α_C_β_C_γ_H_b_H_c_H_d_H_e_H_f_O_a_O_b_O_c_ | 0.00000% |
| C_α_C_β_C_γ_H_b_H_c_H_d_H_e_H_f_O_a_O_b_O_c_ | 0.00000% |
| C_α_C_β_C_γ_H_b_H_c_H_d_H_e_H_f_O_a_O_b_O_c_ | 0.00000% |
| C_α_C_β_C_γ_H_b_H_c_H_d_H_e_H_f_O_a_O_b_O_c_ | 0.00000% |
| C_α_C_β_C_γ_H_b_H_c_H_d_H_e_H_f_O_a_O_b_O_c_ | 0.00000% |
| C_α_C_β_C_γ_H_b_H_c_H_d_H_e_H_f_O_a_O_b_O_c_ | 0.00001% |
| C_α_C_β_C_γ_H_b_H_c_H_d_H_e_H_f_O_a_O_b_O_c_ | 0.00001% |
| sum | 0.02533% |
| **Natural isotopmers of lactate, C_3_H_5_O_3_^-^, that could produce transition *m/z* 91>45** | **The percentage of these natural isotopmers of lactate** |
| C_α_C_β_C_γ_H_b_H_c_H_d_H_e_H_f_O_a_O_b_O_c_ | 0.01124% |
| C_α_C_β_C_γ_H_b_H_c_H_d_H_e_H_f_O_a_O_b_O_c_ | 0.00000% |
| C_α_C_β_C_γ_H_b_H_c_H_d_H_e_H_f_O_a_O_b_O_c_ | 0.00000% |
| C_α_C_β_C_γ_H_b_H_c_H_d_H_e_H_f_O_a_O_b_O_c_ | 0.00000% |
| C_α_C_β_C_γ_H_b_H_c_H_d_H_e_H_f_O_a_O_b_O_c_ | 0.19744% |
| C_α_C_β_C_γ_H_b_H_c_H_d_H_e_H_f_O_a_O_b_O_c_ | 0.00011% |
| C_α_C_β_C_γ_H_b_H_c_H_d_H_e_H_f_O_a_O_b_O_c_ | 0.00011% |
| C_α_C_β_C_γ_H_b_H_c_H_d_H_e_H_f_O_a_O_b_O_c_ | 0.00011% |
| C_α_C_β_C_γ_H_b_H_c_H_d_H_e_H_f_O_a_O_b_O_c_ | 0.00011% |
| C_α_C_β_C_γ_H_b_H_c_H_d_H_e_H_f_O_a_O_b_O_c_ | 0.00011% |
| C_α_C_β_C_γ_H_b_H_c_H_d_H_e_H_f_O_a_O_b_O_c_ | 0.00011% |
| C_α_C_β_C_γ_H_b_H_c_H_d_H_e_H_f_O_a_O_b_O_c_ | 0.00040% |
| C_α_C_β_C_γ_H_b_H_c_H_d_H_e_H_f_O_a_O_b_O_c_ | 0.00040% |
| C_α_C_β_C_γ_H_b_H_c_H_d_H_e_H_f_O_a_O_b_O_c_ | 0.00000% |
| C_α_C_β_C_γ_H_b_H_c_H_d_H_e_H_f_O_a_O_b_O_c_ | 0.00000% |
| C_α_C_β_C_γ_H_b_H_c_H_d_H_e_H_f_O_a_O_b_O_c_ | 0.00000% |
| sum | 0.21017% |
| **Natural isotopmers of lactate, C_3_H_5_O_3_^-^, that could produce transition *m/z* 92>45** | **The percentage of these natural isotopmers of lactate** |
| C_α_C_β_C_γ_H_b_H_c_H_d_H_e_H_f_O_a_O_b_O_c_ | 0.00012% |
| C_α_C_β_C_γ_H_b_H_c_H_d_H_e_H_f_O_a_O_b_O_c_ | 0.00000% |
| C_α_C_β_C_γ_H_b_H_c_H_d_H_e_H_f_O_a_O_b_O_c_ | 0.00000% |
| C_α_C_β_C_γ_H_b_H_c_H_d_H_e_H_f_O_a_O_b_O_c_ | 0.00000% |
| C_α_C_β_C_γ_H_b_H_c_H_d_H_e_H_f_O_a_O_b_O_c_ | 0.00000% |
| C_α_C_β_C_γ_H_b_H_c_H_d_H_e_H_f_O_a_O_b_O_c_ | 0.00000% |
| C_α_C_β_C_γ_H_b_H_c_H_d_H_e_H_f_O_a_O_b_O_c_ | 0.00000% |
| C_α_C_β_C_γ_H_b_H_c_H_d_H_e_H_f_O_a_O_b_O_c_ | 0.00000% |
| C_α_C_β_C_γ_H_b_H_c_H_d_H_e_H_f_O_a_O_b_O_c_ | 0.00000% |
| C_α_C_β_C_γ_H_b_H_c_H_d_H_e_H_f_O_a_O_b_O_c_ | 0.00000% |
| C_α_C_β_C_γ_H_b_H_c_H_d_H_e_H_f_O_a_O_b_O_c_ | 0.00000% |
| C_α_C_β_C_γ_H_b_H_c_H_d_H_e_H_f_O_a_O_b_O_c_ | 0.00000% |
| C_α_C_β_C_γ_H_b_H_c_H_d_H_e_H_f_O_a_O_b_O_c_ | 0.00000% |
| C_α_C_β_C_γ_H_b_H_c_H_d_H_e_H_f_O_a_O_b_O_c_ | 0.00000% |
| C_α_C_β_C_γ_H_b_H_c_H_d_H_e_H_f_O_a_O_b_O_c_ | 0.00000% |
| C_α_C_β_C_γ_H_b_H_c_H_d_H_e_H_f_O_a_O_b_O_c_ | 0.00000% |
| C_α_C_β_C_γ_H_b_H_c_H_d_H_e_H_f_O_a_O_b_O_c_ | 0.00214% |
| C_α_C_β_C_γ_H_b_H_c_H_d_H_e_H_f_O_a_O_b_O_c_ | 0.00000% |
| C_α_C_β_C_γ_H_b_H_c_H_d_H_e_H_f_O_a_O_b_O_c_ | 0.00000% |
| C_α_C_β_C_γ_H_b_H_c_H_d_H_e_H_f_O_a_O_b_O_c_ | 0.00000% |
| C_α_C_β_C_γ_H_b_H_c_H_d_H_e_H_f_O_a_O_b_O_c_ | 0.00000% |
| C_α_C_β_C_γ_H_b_H_c_H_d_H_e_H_f_O_a_O_b_O_c_ | 0.00000% |
| C_α_C_β_C_γ_H_b_H_c_H_d_H_e_H_f_O_a_O_b_O_c_ | 0.00000% |
| C_α_C_β_C_γ_H_b_H_c_H_d_H_e_H_f_O_a_O_b_O_c_ | 0.00000% |
| C_α_C_β_C_γ_H_b_H_c_H_d_H_e_H_f_O_a_O_b_O_c_ | 0.00000% |
| C_α_C_β_C_γ_H_b_H_c_H_d_H_e_H_f_O_a_O_b_O_c_ | 0.00000% |
| C_α_C_β_C_γ_H_b_H_c_H_d_H_e_H_f_O_a_O_b_O_c_ | 0.00000% |
| C_α_C_β_C_γ_H_b_H_c_H_d_H_e_H_f_O_a_O_b_O_c_ | 0.00000% |
| C_α_C_β_C_γ_H_b_H_c_H_d_H_e_H_f_O_a_O_b_O_c_ | 0.00000% |
| C_α_C_β_C_γ_H_b_H_c_H_d_H_e_H_f_O_a_O_b_O_c_ | 0.00000% |
| C_α_C_β_C_γ_H_b_H_c_H_d_H_e_H_f_O_a_O_b_O_c_ | 0.00000% |
| C_α_C_β_C_γ_H_b_H_c_H_d_H_e_H_f_O_a_O_b_O_c_ | 0.00000% |
| C_α_C_β_C_γ_H_b_H_c_H_d_H_e_H_f_O_a_O_b_O_c_ | 0.00000% |
| C_α_C_β_C_γ_H_b_H_c_H_d_H_e_H_f_O_a_O_b_O_c_ | 0.00000% |
| C_α_C_β_C_γ_H_b_H_c_H_d_H_e_H_f_O_a_O_b_O_c_ | 0.00000% |
| C_α_C_β_C_γ_H_b_H_c_H_d_H_e_H_f_O_a_O_b_O_c_ | 0.00000% |
| C_α_C_β_C_γ_H_b_H_c_H_d_H_e_H_f_O_a_O_b_O_c_ | 0.00000% |
| C_α_C_β_C_γ_H_b_H_c_H_d_H_e_H_f_O_a_O_b_O_c_ | 0.00000% |
| C_α_C_β_C_γ_H_b_H_c_H_d_H_e_H_f_O_a_O_b_O_c_ | 0.00000% |
| C_α_C_β_C_γ_H_b_H_c_H_d_H_e_H_f_O_a_O_b_O_c_ | 0.00000% |
| C_α_C_β_C_γ_H_b_H_c_H_d_H_e_H_f_O_a_O_b_O_c_ | 0.00000% |
| C_α_C_β_C_γ_H_b_H_c_H_d_H_e_H_f_O_a_O_b_O_c_ | 0.00000% |
| C_α_C_β_C_γ_H_b_H_c_H_d_H_e_H_f_O_a_O_b_O_c_ | 0.00000% |
| C_α_C_β_C_γ_H_b_H_c_H_d_H_e_H_f_O_a_O_b_O_c_ | 0.00000% |
| C_α_C_β_C_γ_H_b_H_c_H_d_H_e_H_f_O_a_O_b_O_c_ | 0.00000% |
| C_α_C_β_C_γ_H_b_H_c_H_d_H_e_H_f_O_a_O_b_O_c_ | 0.00000% |
| C_α_C_β_C_γ_H_b_H_c_H_d_H_e_H_f_O_a_O_b_O_c_ | 0.00000% |
| C_α_C_β_C_γ_H_b_H_c_H_d_H_e_H_f_O_a_O_b_O_c_ | 0.00000% |
| C_α_C_β_C_γ_H_b_H_c_H_d_H_e_H_f_O_a_O_b_O_c_ | 0.00000% |
| C_α_C_β_C_γ_H_b_H_c_H_d_H_e_H_f_O_a_O_b_O_c_ | 0.00000% |
| C_α_C_β_C_γ_H_b_H_c_H_d_H_e_H_f_O_a_O_b_O_c_ | 0.00000% |
| C_α_C_β_C_γ_H_b_H_c_H_d_H_e_H_f_O_a_O_b_O_c_ | 0.00000% |
| C_α_C_β_C_γ_H_b_H_c_H_d_H_e_H_f_O_a_O_b_O_c_ | 0.00000% |
| C_α_C_β_C_γ_H_b_H_c_H_d_H_e_H_f_O_a_O_b_O_c_ | 0.00000% |
| C_α_C_β_C_γ_H_b_H_c_H_d_H_e_H_f_O_a_O_b_O_c_ | 0.00000% |
| C_α_C_β_C_γ_H_b_H_c_H_d_H_e_H_f_O_a_O_b_O_c_ | 0.00000% |
| C_α_C_β_C_γ_H_b_H_c_H_d_H_e_H_f_O_a_O_b_O_c_ | 0.00000% |
| C_α_C_β_C_γ_H_b_H_c_H_d_H_e_H_f_O_a_O_b_O_c_ | 0.00000% |
| C_α_C_β_C_γ_H_b_H_c_H_d_H_e_H_f_O_a_O_b_O_c_ | 0.00000% |
| C_α_C_β_C_γ_H_b_H_c_H_d_H_e_H_f_O_a_O_b_O_c_ | 0.00000% |
| C_α_C_β_C_γ_H_b_H_c_H_d_H_e_H_f_O_a_O_b_O_c_ | 0.00000% |
| C_α_C_β_C_γ_H_b_H_c_H_d_H_e_H_f_O_a_O_b_O_c_ | 0.00000% |
| C_α_C_β_C_γ_H_b_H_c_H_d_H_e_H_f_O_a_O_b_O_c_ | 0.00000% |
| C_α_C_β_C_γ_H_b_H_c_H_d_H_e_H_f_O_a_O_b_O_c_ | 0.00000% |
| C_α_C_β_C_γ_H_b_H_c_H_d_H_e_H_f_O_a_O_b_O_c_ | 0.00000% |
| C_α_C_β_C_γ_H_b_H_c_H_d_H_e_H_f_O_a_O_b_O_c_ | 0.00000% |
| C_α_C_β_C_γ_H_b_H_c_H_d_H_e_H_f_O_a_O_b_O_c_ | 0.00000% |
| C_α_C_β_C_γ_H_b_H_c_H_d_H_e_H_f_O_a_O_b_O_c_ | 0.00000% |
| C_α_C_β_C_γ_H_b_H_c_H_d_H_e_H_f_O_a_O_b_O_c_ | 0.00000% |
| C_α_C_β_C_γ_H_b_H_c_H_d_H_e_H_f_O_a_O_b_O_c_ | 0.00000% |
| C_α_C_β_C_γ_H_b_H_c_H_d_H_e_H_f_O_a_O_b_O_c_ | 0.00000% |
| C_α_C_β_C_γ_H_b_H_c_H_d_H_e_H_f_O_a_O_b_O_c_ | 0.00000% |
| C_α_C_β_C_γ_H_b_H_c_H_d_H_e_H_f_O_a_O_b_O_c_ | 0.00000% |
| C_α_C_β_C_γ_H_b_H_c_H_d_H_e_H_f_O_a_O_b_O_c_ | 0.00000% |
| C_α_C_β_C_γ_H_b_H_c_H_d_H_e_H_f_O_a_O_b_O_c_ | 0.00000% |
| C_α_C_β_C_γ_H_b_H_c_H_d_H_e_H_f_O_a_O_b_O_c_ | 0.00000% |
| C_α_C_β_C_γ_H_b_H_c_H_d_H_e_H_f_O_a_O_b_O_c_ | 0.00002% |
| C_α_C_β_C_γ_H_b_H_c_H_d_H_e_H_f_O_a_O_b_O_c_ | 0.00002% |
| C_α_C_β_C_γ_H_b_H_c_H_d_H_e_H_f_O_a_O_b_O_c_ | 0.00008% |
| C_α_C_β_C_γ_H_b_H_c_H_d_H_e_H_f_O_a_O_b_O_c_ | 0.00008% |
| sum | 0.00248% |

**Table S4. The percentage of ion transitions derived from natural isotopmers of lactate**

| **ion transition** | **percentage** | **relative percentage** |
| --- | --- | --- |
| **89/43** | 96.08071% | 100% |
| **90/44** | 2.14667% | 2.2% |
| **90/43** | 1.13352% | 1.2% |
| **91/45** | 0.21017% | 0.22% |
| **91/44** | 0.02533% | 0.026% |
| **92/45** | 0.00248% | 0.0026% |

**Table S5. A time course of lactate isotopmers and isotoplogues (mean±SD, n=6) generated by 4T1, Hela, K562 and thymocyte, traced by [1,2-^13^C_2_]glucose.**

|  |  | **lactate** | **[2,3-^13^C_2_]lactate** | **[2-^13^C]- or [3-^13^C]lactate** | **[1-^13^C]lactate** | **[1,2-^13^C_2_]- or [1,3-^13^C_2_]lactate** | **[U-^13^C]lactate** |
| --- | --- | --- | --- | --- | --- | --- | --- |
| **4T1** | **1h** | 44% ± 1.7% | 54% ± 1.7% | 1.2% ± 0.22% | / | 0.21% ± 0.045% | 0.71% ± 0.13% |
|  | **2h** | 42% ± 0.98% | 55% ± 0.97% | 1.4% ± 0.13% | / | 0.31% ± 0.038% | 0.79% ± 0.12% |
|  | **4h** | 40% ± 0.56% | 57% ± 0.65% | 1.5% ± 0.094% | / | 0.38% ± 0.059% | 0.85% ± 0.12% |
|  | **6h** | 40% ± 0.44% | 57% ± 0.53% | 1.7% ± 0.050% | 0.036% ± 0.022% | 0.43% ± 0.062% | 0.88% ± 0.082% |
|  | **8h** | 40% ± 0.36% | 57% ± 0.45% | 1.7% ± 0.049% | 0.053% ± 0.028% | 0.50% ± 0.077% | 0.91% ± 0.087% |
|  | **10h** | 39% ± 0.80% | 57% ± 0.99% | 1.8% ± 0.057% | 0.046% ± 0.030% | 0.56% ± 0.10% | 0.95% ± 0.11% |
|  | **12h** | 39% ± 0.53% | 57% ± 0.69% | 1.9% ± 0.071% | 0.047% ± 0.022% | 0.61% ± 0.090% | 0.96% ± 0.090% |
| **Hela** | **1h** | 44% ± 0.76% | 53% ± 0.70% | 1.7% ± 0.29% | / | 0.40% ± 0.057% | 0.97% ± 0.12% |
|  | **2h** | 41% ± 0.44% | 55% ± 0.45% | 1.9% ± 0.14% | 0.039% ± 0.038% | 0.45% ± 0.032% | 1.0% ± 0.068% |
|  | **4h** | 40% ± 0.40% | 56% ± 0.50% | 1.9% ± 0.22% | 0.051% ± 0.030% | 0.46% ± 0.044% | 1.0% ± 0.089% |
|  | **6h** | 40% ± 0.22% | 56% ± 0.20% | 1.9% ± 0.19% | 0.029% ± 0.029% | 0.51% ± 0.026% | 1.1% ± 0.071% |
|  | **8h** | 40% ± 0.20% | 56% ± 0.31% | 2.1% ± 0.20% | 0.041% ± 0.030% | 0.57% ± 0.029% | 1.2% ± 0.076% |
|  | **10h** | 40% ± 0.25% | 56% ± 0.32% | 2.2% ± 0.21% | 0.058% ± 0.025% | 0.65% ± 0.034% | 1.3% ± 0.086% |
|  | **12h** | 40% ± 0.42% | 55% ± 0.49% | 2.3% ± 0.20% | 0.079% ± 0.031% | 0.70% ± 0.020% | 1.4% ± 0.050% |
| **K562** | **1h** | 44% ± 1.0% | 53% ± 0.86% | 1.5% ± 0.17% | / | 0.36% ± 0.076% | 0.96% ± 0.078% |
|  | **2h** | 41% ± 0.58% | 56% ± 0.66% | 1.8% ± 0.088% | / | 0.51% ± 0.039% | 1.1% ± 0.088% |
|  | **4h** | 41% ± 0.30% | 56% ± 0.25% | 1.8% ± 0.10% | / | 0.62% ± 0.027% | 1.2% ± 0.050% |
|  | **6h** | 40% ± 0.43% | 56% ± 0.46% | 2.0% ± 0.062% | 0.073% ± 0.026% | 0.75% ± 0.048% | 1.1% ± 0.073% |
|  | **8h** | 40% ± 0.33% | 56% ± 0.33% | 2.1% ± 0.090% | 0.071% ± 0.012% | 0.80% ± 0.038% | 1.2% ± 0.061% |
|  | **10h** | 40% ± 0.31% | 56% ± 0.31% | 2.2% ± 0.078% | 0.072% ± 0.023% | 0.84% ± 0.040% | 1.2% ± 0.025% |
|  | **12h** | 39% ± 0.47% | 56% ± 0.48% | 2.1% ± 0.067% | 0.079% ± 0.024% | 0.86% ± 0.037% | 1.2% ± 0.042% |
| **thymocyte** | **1h** | 56% ± 2.0% | 42% ± 2.1% | 1.0% ± 0.080% | 0.12% ± 0.090% | 0.20% ± 0.038% | 0.60% ± 0.092% |
|  | **2h** | 48% ± 0.75% | 50% ± 0.82% | 1.2% ± 0.10% | 0.088% ± 0.078% | 0.22% ± 0.029% | 0.66% ± 0.043% |
|  | **4h** | 44% ± 0.39% | 54% ± 0.42% | 1.2% ± 0.072% | 0.090% ± 0.048% | 0.23% ± 0.028% | 0.75% ± 0.029% |
|  | **6h** | 43% ± 0.27% | 55% ± 0.32% | 1.2% ± 0.068% | 0.10% ± 0.032% | 0.25% ± 0.018% | 0.82% ± 0.047% |
|  | **8h** | 42% ± 0.46% | 55% ± 0.39% | 1.2% ± 0.061% | 0.12% ± 0.028% | 0.27% ± 0.023% | 0.86% ± 0.043% |
|  | **10h** | 42% ± 0.28% | 56% ± 0.25% | 1.3% ± 0.068% | 0.12% ± 0.022% | 0.27% ± 0.025% | 0.89% ± 0.037% |
|  | **12h** | 42% ± 0.28% | 56% ± 0.30% | 1.3% ± 0.074% | 0.13% ± 0.027% | 0.28% ± 0.022% | 0.93% ± 0.030% |

4T1, Hela, K562 and thymocyte were incubated in serum-free RPMI-1640 medium containing 6 mM [1,2-^13^C_2_]glucose in a humidified CO_2_ incubator and culture supernatant was collected at every time point for LC-MS analysis as described in Materials and Methods, the time-course experiment illustrate that a steady-state generation of lactate isotopologues attained 4 hours after incubation and maintained thereafter. Data are mean ± SD, n=12, from 2 independent experiments.

**Table S6. The percentage of lactate isotopomers and isotopologues (mean±SD, n=6) generated by 4T1 and Hela, cultured with or without FBS, traced by [1-^13^C]-, [6-^13^C]-, [1,2-^13^C_2_]- and [1,2,3-^13^C_3_]glucose.**

|  |  |  | **lactate** | **[2-^13^C]- or [3-^13^C]lactate** | **[1-^13^C]lactate** | **[1,2-^13^C_2_]- or [1,3-^13^C_2_]lactate** | **[2,3-^13^C_2_]lactate** | **[U-^13^C]lactate** |
| --- | --- | --- | --- | --- | --- | --- | --- | --- |
| **4T1** | **[1-^13^C]glucose** | **+FBS** | 48% ± 1.6% | 51% ± 1.5% | 0.16% ± 0.015% | 0.72% ± 0.016% | 0.65% ± 0.068% | / |
|  |  | **-FBS** | 48% ± 0.31% | 51% ± 0.29% | 0.18% ± 0.019% | 0.76% ± 0.0082% | 0.61% ± 0.068% | / |
|  | **[6-^13^C]glucose** | **+FBS** | 46% ± 1.2% | 52% ± 1.2% | 0.11% ± 0.011% | 0.67% ± 0.016% | 0.64% ± 0.18% | / |
|  |  | **-FBS** | 45% ± 0.34% | 53% ± 0.35% | 0.071% ± 0.018% | 0.68% ± 0.019% | 0.77% ± 0.083% | / |
|  | **[1,2-^13^C_2_]glucose** | **+FBS** | 41% ± 0.63% | 2.1% ± 0.12% | 0.12% ± 0.017% | 0.34% ± 0.044% | 56% ± 0.49% | 0.88% ± 0.038% |
|  |  | **-FBS** | 41% ± 0.20% | 2.3% ± 0.10% | 0.093% ± 0.0099% | 0.61% ± 0.083% | 55% ± 0.34% | 1.0% ± 0.033% |
|  | **[1,2,3-^13^C_3_]glucose** | **+FBS** | 42% ± 1.7% | 0.42% ± 0.060% | 0.20% ± 0.053% | 0.12% ± 0.030% | 0.29% ± 0.063% | 57% ± 1.5% |
|  |  | **-FBS** | 43% ± 0.28% | 0.32% ± 0.46% | 0.080% ± 0.012% | 0.15% ± 0.031% | 0.18% ± 0.0091% | 57% ± 0.27% |
| **Hela** | **[1-^13^C]glucose** | **+FBS** | 51% ± 0.42% | 47% ± 0.47% | 0.28% ± 0.016% | 0.84% ± 0.041% | 0.64% ± 0.023% | / |
|  |  | **-FBS** | 50 % ± 1.2% | 48% ± 1.2% | 0.28% ± 0.023% | 0.90% ± 0.036% | 0.68% ± 0.040% | / |
|  | **[6-^13^C]glucose** | **+FBS** | 48% ± 0.39% | 51% ± 0.39% | 0.13% ± 0.015% | 0.65% ± 0.0079% | 0.68% ± 0.017% | / |
|  |  | **-FBS** | 48% ± 2.8% | 50% ± 2.9% | 0.20% ± 0.10% | 0.81% ± 0.12% | 0.69% ± 0.040% | / |
|  | **[1,2-^13^C_2_]glucose** | **+FBS** | 42% ± 0.91% | 2.8% ± 0.14% | 0.22% ± 0.037% | 0.63% ± 0.028% | 53% ± 1.1% | 1.4% ± 0.087% |
|  |  | **-FBS** | 42% ± 0.35% | 3.1% ± 0.17% | 0.15% ± 0.029% | 0.92% ± 0.051% | 52% ± 0.69% | 1.6% ± 0.13% |
|  | **[1,2,3-^13^C_3_]glucose** | **+FBS** | 45% ± 0.76% | 0.42% ± 0.022% | 0.54% ± 0.063% | 0.19% ± 0.015% | 0.85% ± 0.019% | 53% ± 0.84% |
|  |  | **-FBS** | 43% ± 0.30% | 0.35% ± 0.037% | 0.13% ± 0.028% | 0.16% ± 0.019% | 0.25% ± 0.025% | 56% ± 0.38% |

4T1 and Hela were incubated in RPMI-1640 medium with 10% fetal bovine serum or not, containing 6 mM [1-^13^C]-, [6-^13^C]-, [1,2-^13^C_2_]- and [1,2,3-^13^C_3_]glucose for 12 hours in a humidified CO_2_ incubator and culture supernatant was collected for LC-MS/MS analysis as described in Materials and Methods, the metabolic pattern of cells does not change whether the medium contains fetal bovine serum or not. Data are mean ± SD, n=12, from 2 independent experiments.

**Supplementary Table S7. The percentage of lactate isotopomers and isotopologues (mean±SD, n=12) generated by Hela, K562, and thymocytes, traced by [1-^13^C]glucose (upper part), the percentage of lactate (mean±SD, n=12) derived from glycolysis, PPP and other sources (down part).**

|  | **[2-^13^C]- [3-^13^C]lactate** | **lactate** | **[1,2-^13^C_2_]- or [1,3-^13^C_2_]lactate** | **[1-^13^C]lactate** | **[U-^13^C]lactate** | **[2,3-^13^C_2_]lactate** | **Total generated Lactate** |
| --- | --- | --- | --- | --- | --- | --- | --- |
| **Hela** | 48% ± 0.63% | 50% ± 0.62% | 0.97% ± 0.031% | 0.23% ± 0.043% | 0.028% ± 0.0052% | 0.91% ± 0.030% | 41 ± 3.3  (μmol/million cell/12h) |
| **K562** | 50% ± 0.32% | 48% ± 0.35% | 0.80% ± 0.026% | 0.19% ± 0.021% | / | 0.90% ± 0.039% | 8.1 ± 0.37  (μmol/million cell/12h) |
| **Thymocytes** | 46% ± 0.53% | 52% ± 0.47% | 0.93% ± 0.050% | 0.23% ± 0.042% | / | 0.82% ± 0.069% | 63 ± 1.4  (nmol/million cell/12h) |

|  | **Lactate derived from glycolysis** | | **Lactate derived from nonoxidative PPP** | | **Lactate derived from other sources** |
| --- | --- | --- | --- | --- | --- |
|  | Glc carbon 1,2,3 | Glc carbon 4,5,6 | Glc carbon 2,3 | Glc carbon 4,5,6 |  |
|  | [3-^13^C]lactate | lactate | lactate | lactate | lactate |
| **Hela** | 48% ± 0.63% | 37% ± 0.58% | 4.3% ± 0.0% | 6.5% ± 0.0% | 2.3% ± 0.093% |
| **sum** | 85% | | 11% | |  |
| **K562** | 50% ± 0.32% | 37% ± 0.42% | 3.9% ± 0.0% | 5.9% ± 0.0% | 1.5% ± 0.12% |
| **sum** | 87% | | 9.8% | |  |
| **Thymocytes** | 46% ± 0.53% | 36% ± 0.58% | 3.2% ± 0.0% | 4.9% ± 0.0% | 7.9% ± 0.19% |
| **sum** | 82% | | 8.1% | |  |

Hela, K562 and thymocyte were incubated in serum-free RPMI-1640 medium containing 6 mM [1-^13^C]glucose for 12 hours in a humidified CO_2_ incubator and culture supernatant was collected for LC-MS analysis as described in Materials and Methods. The upper part summarizes the percentage of all lactate isotopologues. The down part assigned the percentage of isotopologues that are generated from glycolysis, pentose phosphate pathway, and other sources. Specifically, the percentage of lactate is composed of three parts, from glycolysis, pentose phosphate pathway (calculated based on percentage of [2-^13^C]lactate and [1,2-^13^C_2_]lactate in Table 3), and other sources (according to percentage listed in Table 1). Data are mean ± SD, n=12, from 2 independent experiments.

**Supplementary Table S8. The percentage of lactate isotopomers and isotopologues (mean ± SD, n=12) generated by Hela, K562, and thymocytes, traced by [6-^13^C]glucose (upper part), the percentage of lactate (mean ± SD, n=12) derived from glycolysis, PPP and other sources (down part).**

|  | **lactate** | **[2-^13^C]- or [3-^13^C]lactate** | **[1-^13^C]lactate** | **[U-^13^C]lactate** | **[2,3-^13^C_2_]lactate** | **[1,2-^13^C_2_]- or [1,3-^13^C_2_]lactate** | **Total generated Lactate** |
| --- | --- | --- | --- | --- | --- | --- | --- |
| **Hela** | 46% ± 0.53% | 52% ± 0.52% | 0.060% ± 0.020% | 0.027% ± 0.0054% | 0.94% ± 0.032% | 0.74% ± 0.031% | 41 ± 3.3  (μmol/million cell/12h) |
| **K562** | 45% ± 0.27% | 53% ± 0.27% | 0.045% ± 0.015% | / | 0.92% ± 0.043% | 0.73% ± 0.024% | 7.7 ± 0.50  (μmol/million cell/12h) |
| **Thymocytes** | 50% ± 0.40% | 49% ± 0.36% | 0.14% ± 0.036% | / | 0.86% ± 0.065% | 0.66% ± 0.026% | 63 ± 1.7  (nmol/million cell/12h) |

|  | **Lactate derived from glycolysis** | | **Lactate derived from nonoxidative PPP** | | **Lactate derived from other sources** |
| --- | --- | --- | --- | --- | --- |
|  | Glc carbon 1,2,3 | Glc carbon 4,5,6 | Glc carbon 2,3 | Glc carbon 4,5,6 |  |
|  | lactate | [3-^13^C]lactate | lactate | [3-^13^C]lactate | lactate |
| **Hela** | 39% ± 0.60% | 46% ± 0.52% | 4.3% ± 0.00% | 6.5% ± 0.00% | 2.3% ± 0.093% |
| **sum** | 85% | | 11% | |  |
| **K562** | 40% ± 0.30% | 47% ± 0.27% | 4.0% ± 0.00% | 5.9% ± 0.00% | 1.5% ± 0.12% |
| **sum** | 87% | | 9.9% | |  |
| **Thymocytes** | 38% ± 0.47% | 44% ± 0.36% | 3.2% ± 0.00% | 4.9% ± 0.00% | 7.9% ± 0.19% |
| **sum** | 83% | | 8.1% | |  |

Hela, K562 and thymocyte were incubated in serum-free RPMI-1640 medium containing 6 mM [6-^13^C]glucose for 12 hours in a humidified CO_2_ incubator and culture supernatant was collected for LC-MS analysis as described in Materials and Methods. The upper part summarizes the percentage of all lactate isotopologues. The down part assigned the percentage of isotopologues that are generated from glycolysis, pentose phosphate pathway, and other sources. Specifically, the percentage of lactate is composed of three parts, from glycolysis, pentose phosphate pathway (calculated based on percentage of [2-^13^C]lactate and [1,2-^13^C_2_]lactate in Table 3), and other sources (according to percentage listed in Table 1). Data are mean ± SD, n=12, from 2 independent experiments.

**Supplementary Table S9. The percentage of lactate isotopomers and isotopologues (mean ± SD, n=12) generated by Hela, K562, and thymocytes, traced by [1,2,3-^13^C_3_]glucose (upper part), the percentage of lactate (mean ± SD, n=12) derived from glycolysis, PPP and other sources (down part).**

|  | **[U-^13^C]lactate** | **lactate** | **[2-^13^C]- or [3-^13^C]lactate** | **[1,2-^13^C_2_]- or [1,3-^13^C_2_]lactate** | **[1-^13^C]lactate** | **[2,3-^13^C_2_]lactate** | **Total generated Lactate** |
| --- | --- | --- | --- | --- | --- | --- | --- |
| **Hela** | 57% ± 0.56% | 42% ± 0.43% | 0.34% ± 0.058% | 0.19% ± 0.061% | 0.059% ± 0.029% | 0.27% ± 0.034% | 39 ± 3.1  (μmol/million cell/12h) |
| **K562** | 57% ± 0.47% | 42% ± 0.49% | 0.30% ± 0.041% | 0.14% ± 0.024% | 0.034% ± 0.021% | 0.25% ± 0.030% | 8.2 ± 0.74  (μmol/million cell/12h) |
| **Thymocytes** | 52% ± 0.33% | 46% ± 0.35% | 0.40% ± 0.088% | 0.27% ± 0.062% | 0.41% ± 0.049% | 0.91% ± 0.064% | 57 ± 3.0  (nmol/million cell/12h) |

|  | **Lactate derived from glycolysis** | | **Lactate derived from nonoxidative PPP** | | **Lactate derived from other sources** |
| --- | --- | --- | --- | --- | --- |
|  | Glc carbon 1,2,3 | Glc carbon 4,5,6 | Glc carbon 2,3 | Glc carbon 4,5,6 |  |
|  | [1,2,3-^13^C_3_]lactate | lactate | [1,2,3-^13^C_3_]lactate | lactate | lactate |
| **Hela** | 52% ± 0.56% | 34% ± 0.39% | 4.3% ± 0.00% | 6.5% ± 0.00% | 2.3% ± 0.093% |
| **sum** | 86% | | 11% | |  |
| **K562** | 53% ± 0.47% | 35% ± 0.53% | 4.0% ± 0.00% | 5.9% ± 0.00% | 1.5% ± 0.12% |
| **sum** | 88% | | 9.9% | |  |
| **Thymocytes** | 49% ± 0.33% | 33% ± 0.46% | 3.2% ± 0.00% | 4.9% ± 0.00% | 7.9% ± 0.19% |
| **sum** | 82% | | 8.1% | |  |

Hela, K562 and thymocyte were incubated in serum-free RPMI-1640 medium containing 6 mM [1,2,3-^13^C_3_]glucose for 12 hours in a humidified CO_2_ incubator and culture supernatant was collected for LC-MS analysis as described in Materials and Methods. The upper part summarizes the percentage of all lactate isotopologues. The down part assigned the percentage of isotopologues that are generated from glycolysis, pentose phosphate pathway, and other sources. Specifically, the percentage of lactate is composed of three parts, from glycolysis, pentose phosphate pathway (calculated based on percentage of [2-^13^C]lactate and [1,2-^13^C_2_]lactate in Table 3), and other sources (according to percentage listed in Table 1). Data are mean ± SD, n=12, from 2 independent experiments.

**Supplementary Table S10. The percentage of lactate isotopomers and isotopologues (mean±SD, n=12) generated by Hela, K562, and thymocytes, traced by [4-^13^C]glucose (upper part), the percentage of lactate (mean±SD, n=12) derived from glycolysis, PPP and other sources (down part).**

|  | **lactate** | **[1-^13^C]lactate** | **[3-^13^C]lactate** | **[1,2-^13^C_2_]- or [1,3-^13^C_2_]lactate** | **[U-^13^C]lactate** | **[2,3-^13^C_2_]lactate** | **Total generated Lactate** |
| --- | --- | --- | --- | --- | --- | --- | --- |
| **Hela** | 50% ± 0.19% | 48% ± 0.19% | 0.45% ± 0.054% | 1.5% ± 0.070% | 0.15% ± 0.016% | 0.069% ± 0.016% | 39 ± 3.4  (μmol/million cell/12) |
| **K562** | 49% ± 0.50% | 49% ± 0.50% | 0.44% ± 0.046% | 1.4% ± 0.044% | 0.17% ± 0.019% | 0.067% ± 0.0093% | 8.1 ± 0.57  (μmol/million cell/12h) |
| **Thymocytes** | 54% ± 0.24% | 44% ± 0.28% | 0.54% ± 0.049% | 1.3% ± 0.052% | 0.14% ± 0.021% | 0.086% ± 0.023% | 60 ± 1.5  (nmol/million cell/12h) |

|  | **Lactate derived from glycolysis** | | **Lactate derived from nonoxidative PPP** | | **Lactate derived from other sources** |
| --- | --- | --- | --- | --- | --- |
|  | Glc carbon 1,2,3 | Glc carbon 4,5,6 | Glc carbon 2,3 | Glc carbon 4,5,6 |  |
|  | lactate | [1-^13^C]lactate | lactate | [1-^13^C]lactate | lactate |
| **Hela** | 43% ± 0.23% | 41% ± 0.19% | 4.3% ± 0.0% | 6.5% ± 0.0% | 2.3% ± 0.093% |
| **sum** | 84% | | 11% | |  |
| **K562** | 44% ± 0.48% | 43% ± 0.50% | 4.0% ± 0.0% | 5.9% ± 0.0% | 1.5% ± 0.12% |
| **sum** | 87% | | 9.9% | |  |
| **Thymocytes** | 43% ± 0.32% | 39% ± 0.28% | 3.2% ± 0.0% | 4.9% ± 0.0% | 7.9% ± 0.19% |
| **sum** | 82% | | 8.1% | |  |

Hela, K562 and thymocyte were incubated in serum-free RPMI-1640 medium containing 6 mM [4-^13^C]glucose for 12 hours in a humidified CO_2_ incubator and culture supernatant was collected for LC-MS analysis as described in Materials and Methods. The upper part summarizes the percentage of all lactate isotopologues. The down part assigned the percentage of isotopologues that are generated from glycolysis, pentose phosphate pathway, and other sources. Specifically, the percentage of lactate is composed of three parts, from glycolysis, pentose phosphate pathway (calculated based on percentage of [2-^13^C]lactate and [1,2-^13^C_2_]lactate in Table 3), and other sources (according to percentage listed in Table 1). Data are mean ± SD, n=12, from 2 independent experiments.

**Supplementary Table S11.** Standards of [2,3-^13^C_2_]lactate mixed with lactate, and [2,3-^13^C_2_]lactate/lactate concentration ratios were: 9.0, 4.0, 2.3, 1.5, 1.0, 0.67, 0.43, 0.25, and 0.11. These mixtures were analyzed by LC-MS/MS under MRM mode in losing CO and losing H_2_O, as well as analyzed by LC-MS under Q1 multiple ion mode.

**MRM mode in losing CO**

|  | Peak area percentage | |  |  |
| --- | --- | --- | --- | --- |
| [2,3-^13^C_2_]lactate/lactate  concentration ratio | [2,3-^13^C_2_]lactate  (*m/z* 91>45) | Lactate  (*m/z* 89>43) | [2,3-^13^C_2_]lactate/lactate  peak area ratio | [2,3-^13^C_2_]lactate/lactate peak area ratio  [2,3-^13^C_2_]lactate/lactate concentration ratio |
| 9.0 | 93% ± 0.24% | 7.2% ± 0.24% | 13 ± 0.45 | 1.4 ± 0.050 |
| 4.0 | 86% ± 0.61% | 14% ± 0.61% | 6.0 ± 0.29 | 1.5 ± 0.073 |
| 2.3 | 78% ± 0.37% | 22% ± 0.37% | 3.6 ± 0.077 | 1.5 ± 0.033 |
| 1.5 | 69% ± 0.41% | 31% ± 0.41% | 2.2 ± 0.042 | 1.5 ± 0.028 |
| 1.0 | 60% ± 1.1% | 40% ± 1.1% | 1.5 ± 0.070 | 1.5 ± 0.070 |
| 0.67 | 50% ± 0.45% | 50% ± 0.45% | 1.0 ± 0.018 | 1.5 ± 0.027 |
| 0.43 | 39% ± 0.43% | 61% ± 0.43% | 0.63 ± 0.011 | 1.5 ± 0.026 |
| 0.25 | 27% ± 0.20% | 73% ± 0.20% | 0.37 ± 0.0037 | 1.5 ± 0.015 |
| 0.11 | 14% ± 0.21% | 86% ± 0.21% | 0.17 ± 0.0029 | 1.5 ± 0.0026 |
|  | | | | Overall: 1.5 ± 0.050 |

**Q1 multiple ion mode**

|  | Peak area percentage | |  |  |
| --- | --- | --- | --- | --- |
| [2,3-^13^C_2_]lactate/lactate  concentration ratio | [2,3-^13^C_2_]lactate  (*m/z* 91) | Lactate  (*m/z* 89) | [2,3-^13^C_2_]lactate/lactate  peak area ratio | [2,3-^13^C_2_]lactate/lactate peak area ratio  [2,3-^13^C_2_]lactate/lactate concentration ratio |
| 9.0 | 89% ± 0.33% | 11% ± 0.33% | 8.1 ± 0.26 | 0.90 ± 0.030 |
| 4.0 | 78% ± 0.75% | 22% ± 0.75% | 3.7 ± 0.066 | 0.91 ± 0.040 |
| 2.3 | 69% ± 0.46% | 31% ± 0.46% | 2.2 ± 0.048 | 0.96 ± 0.020 |
| 1.5 | 52% ± 0.95% | 48% ± 0.97% | 1.4 ± 0.041 | 0.93 ± 0.038 |
| 1.0 | 50% ± 1.0% | 50% ± 1.0% | 1.0 ± 0.040 | 1.0 ± 0.040 |
| 0.67 | 40% ± 0.72% | 60% ± 0.72% | 0.67 ± 0.020 | 1.0 ± 0.030 |
| 0.43 | 30% ± 0.52% | 70% ± 0.52% | 0.44 ± 0.011 | 1.0 ± 0.025 |
| 0.25 | 21% ± 1.0% | 79% ± 1.0% | 0.27 ± 0.016 | 1.1 ± 0.065 |
| 0.11 | 11% ± 0.56% | 89% ± 0.56% | 0.12 ± 0.0070 | 1.1 ± 0.063 |
|  | | | | Overall: 1.0 ± 0.078 |

**MRM mode in losing H_2_O**

|  | Peak area percentage | |  |  |
| --- | --- | --- | --- | --- |
| [2,3-^13^C_2_]lactate/lactate  concentration ratio | [2,3-^13^C_2_]lactate  (*m/z* 91>73) | Lactate  (*m/z* 89>71) | [2,3-^13^C_2_]lactate/lactate  peak area ratio | [2,3-^13^C_2_]lactate/lactate peak area ratio  [2,3-^13^C_2_]lactate/lactate concentration ratio |
| 9.0 | 91% ± 0.29% | 9.3% ± 0.29% | 9.7 ± 0.32 | 1.1 ± 0.036 |
| 4.0 | 82% ± 0.22% | 18% ± 0.22% | 4.4 ± 0.066 | 1.1 ± 0.016 |
| 2.3 | 72% ± 0.61% | 28% ± 0.61% | 2.6 ± 0.080 | 1.1 ± 0.034 |
| 1.5 | 62% ± 0.30% | 38% ± 0.30% | 1.6 ± 0.021 | 1.1 ± 0.014 |
| 1.0 | 52% ± 0.95% | 48% ± 0.95% | 1.1 ± 0.041 | 1.1 ± 0.041 |
| 0.67 | 43% ± 0.36% | 57% ± 0.36% | 0.74 ± 0.011 | 1.1 ± 0.016 |
| 0.43 | 32% ± 0.31% | 68% ± 0.31% | 0.48 ± 0.0068 | 1.1 ± 0.016 |
| 0.25 | 21% ± 0.34% | 79% ± 0.34% | 0.27 ± 0.0055 | 1.1 ± 0.022 |
| 0.11 | 11% ± 0.16% | 89% ± 0.16% | 0.17 ± 0.0029 | 1.1 ± 0.018 |
|  | | | | Overall: 1.1 ± 0.030 |

**Supplementary Table S12.** Standards of [1-^13^C]lactate mixed with lactate, and [1-^13^C]lactate/lactate concentration ratios were: 9.0, 4.0, 2.3, 1.5, 1.0, 0.67, 0.43, 0.25, and 0.11. These mixtures were analyzed by LC-MS/MS under MRM mode in losing CO and losing H_2_O, as well as analyzed by LC-MS under Q1 multiple ion mode.

**MRM mode in losing CO**

|  | Peak area percentage | |  |  |
| --- | --- | --- | --- | --- |
| [1-^13^C]lactate/lactate  concentration ratio | [1-^13^C]lactate  (*m/z* 90>43) | Lactate  (*m/z* 89>43) | [1-^13^C]lactate/lactate  peak area ratio | [1-^13^C]lactate/lactate peak area ratio  [1-^13^C]lactate/lactate concentration ratio |
| 9.0 | 90% ± 0.34% | 10% ± 0.34% | 8.6 ± 0.32 | 0.96 ± 0.035 |
| 4.0 | 79% ± 0.38% | 21% ± 0.38% | 3.9 ± 0.089 | 0.97 ± 0.022 |
| 2.3 | 69% ± 0.18% | 31% ± 0.18% | 2.3 ± 0.019 | 0.97 ± 0.0082 |
| 1.5 | 60% ± 0.60% | 40% ± 0.60% | 1.5 ± 0.037 | 0.99 ± 0.024 |
| 1.0 | 51% ± 0.36% | 49% ± 0.36% | 1.0 ± 0.015 | 1.0 ± 0.015 |
| 0.67 | 41% ± 0.41% | 59% ± 0.41% | 0.69 ± 0.012 | 1.0 ± 0.018 |
| 0.43 | 31% ± 0.25% | 69% ± 0.25% | 0.45 ± 0.0053 | 1.0 ± 0.012 |
| 0.25 | 21% ± 0.30% | 79% ± 0.30% | 0.26 ± 0.0047 | 1.0 ± 0.019 |
| 0.11 | 9.9% ± 0.26% | 90% ± 0.26% | 0.11 ± 0.0032 | 0.99 ± 0.029 |
|  | | | | Overall: 1.0 ± 0.040 |

**Q1 multiple ion mode**

|  | Peak area percentage | |  |  |
| --- | --- | --- | --- | --- |
| [1-^13^C]lactate/lactate  concentration ratio | [1-^13^C]lactate  (*m/z* 90) | Lactate  (*m/z* 89) | [1-^13^C]lactate/lactate  peak area ratio | [1-^13^C]lactate/lactate peak area ratio  [1-^13^C]lactate/lactate concentration ratio |
| 9.0 | 90% ± 0.38% | 10% ± 0.38% | 8.7 ± 0.35 | 0.97 ± 0.039 |
| 4.0 | 80% ± 0.69% | 20% ± 0.69% | 4.0 ± 0.18 | 1.0 ± 0.045 |
| 2.3 | 70% ± 1.4% | 30% ± 1.4% | 2.3 ± 0.15 | 1.0 ± 0.065 |
| 1.5 | 58% ± 0.95% | 42% ± 0.95% | 1.4 ± 0.055 | 0.93 ± 0.036 |
| 1.0 | 50% ± 1.3% | 50% ± 1.3% | 0.99 ± 0.051 | 0.99 ± 0.051 |
| 0.67 | 39% ± 1.3% | 61% ± 1.3% | 0.64 ± 0.035 | 0.95 ± 0.052 |
| 0.43 | 28% ± 0.51% | 72% ± 0.51% | 0.39 ± 0.010 | 0.91 ± 0.023 |
| 0.25 | 18% ± 0.27% | 82% ± 0.27% | 0.23 ± 0.0042 | 0.91 ± 0.017 |
| 0.11 | 9.1% ± 0.86% | 91% ± 0.86% | 0.10 ± 0.010 | 0.91 ± 0.094 |
|  | | | | Overall: 0.95 ± 0.063 |

**MRM mode in losing H_2_O**

|  | Peak area percentage | |  |  |
| --- | --- | --- | --- | --- |
| [1-^13^C]lactate/lactate  concentration ratio | [1-^13^C]lactate  (*m/z* 90>72) | Lactate  (*m/z* 89>71) | [1-^13^C]lactate/lactate  peak area ratio | [1-^13^C]lactate/lactate peak area ratio  [1-^13^C]lactate/lactate concentration ratio |
| 9.0 | 90% ± 0.32% | 9.7% ± 0.32% | 9.3 ± 0.34 | 1.0 ± 0.038 |
| 4.0 | 80% ± 0.48% | 20% ± 0.48% | 4.0 ± 0.12 | 1.0 ± 0.030 |
| 2.3 | 71% ± 0.69% | 29% ± 0.69% | 2.4 ± 0.079 | 1.0 ± 0.034 |
| 1.5 | 61% ± 0.48% | 39% ± 0.48% | 1.6 ± 0.032 | 1.0 ± 0.021 |
| 1.0 | 51% ± 0.96% | 49% ± 0.96% | 1.1 ± 0.040 | 1.1 ± 0.040 |
| 0.67 | 42% ± 1.1% | 58% ± 1.1% | 0.73 ± 0.033 | 1.1 ± 0.050 |
| 0.43 | 32% ± 0.75% | 68% ± 0.75% | 0.48 ± 0.017 | 1.1 ± 0.039 |
| 0.25 | 21% ± 1.1% | 79% ± 1.1% | 0.27 ± 0.018 | 1.1 ± 0.074 |
| 0.11 | 11% ± 0.76% | 89% ± 0.76% | 0.12 ± 0.0094 | 1.1 ± 0.085 |
|  | | | | Overall: 1.1 ± 0.060 |

**Supplementary Table S13.** Standards of [3-^13^C]lactate mixed with lactate, and [3-^13^C]lactate/lactate concentration ratios were: 9.0, 4.0, 2.3, 1.5, 1.0, 0.67, 0.43, 0.25, and 0.11. These mixtures were analyzed by LC-MS/MS under MRM mode in losing CO and losing H_2_O, as well as analyzed by LC-MS under Q1 multiple ion mode.

**MRM mode in losing CO**

|  | Peak area percentage | |  |  |
| --- | --- | --- | --- | --- |
| [3-^13^C]lactate/lactate  concentration ratio | [3-^13^C]lactate  (*m/z* 90>44) | Lactate  (*m/z* 89>43) | [3-^13^C]lactate/lactate  peak area ratio | [3-^13^C]lactate/lactate peak area ratio  [3-^13^C]lactate/lactate concentration ratio |
| 9.0 | 91% ± 0.38% | 8.9% ± 0.38% | 10 ± 0.48 | 1.1 ± 0.054 |
| 4.0 | 83% ± 0.59% | 17% ± 0.59% | 4.7 ± 0.19 | 1.2 ± 0.049 |
| 2.3 | 74% ± 0.81% | 26% ± 0.81% | 2.8 ± 0.11 | 1.2 ± 0.049 |
| 1.5 | 64% ± 0.81% | 36% ± 0.81% | 1.8 ± 0.065 | 1.2 ± 0.043 |
| 1.0 | 55% ± 0.92% | 45% ± 0.92% | 1.2 ± 0.045 | 1.2 ± 0.045 |
| 0.67 | 46% ± 0.90% | 54% ± 0.90% | 0.84 ± 0.030 | 1.3 ± 0.045 |
| 0.43 | 35% ± 0.62% | 65% ± 0.62% | 0.54 ± 0.015 | 1.3 ± 0.034 |
| 0.25 | 24% ± 0.79% | 76% ± 0.79% | 0.31 ± 0.014 | 1.2 ± 0.054 |
| 0.11 | 12% ± 0.42% | 88% ± 0.42% | 0.14 ± 0.0055 | 1.3 ± 0.050 |
|  |  |  |  | Overall: 1.2 ± 0.073 |

**Q1 multiple ion mode**

|  | Peak area percentage | |  |  |
| --- | --- | --- | --- | --- |
| [3-^13^C]lactate/lactate  concentration ratio | [3-^13^C]lactate  (*m/z* 90) | Lactate  (*m/z* 89) | [3-^13^C]lactate/lactate  peak area ratio | [3-^13^C]lactate/lactate peak area ratio  [3-^13^C]lactate/lactate concentration ratio |
| 9.0 | 89% ± 0.61% | 11% ± 0.61% | 7.7 ± 0.45 | 0.86 ± 0.050 |
| 4.0 | 78% ± 0.80% | 22% ± 0.80% | 3.5 ± 0.16 | 0.89 ± 0.041 |
| 2.3 | 68% ± 1.3% | 32% ± 1.3% | 2.1 ± 0.13 | 0.89 ± 0.054 |
| 1.5 | 58% ± 1.2% | 42% ± 1.2% | 1.4 ± 0.070 | 0.92 ± 0.046 |
| 1.0 | 47% ± 0.92% | 53% ± 0.92% | 0.89 ± 0.023 | 0.89 ± 0.023 |
| 0.67 | 39% ± 0.60% | 61% ± 0.60% | 0.63 ± 0.016 | 0.95 ± 0.024 |
| 0.43 | 29% ± 0.40% | 71% ± 0.40% | 0.40 ± 0.0078 | 0.93 ± 0.018 |
| 0.25 | 19% ± 0.46% | 81% ± 0.46% | 0.23 ± 0.0070 | 0.94 ± 0.028 |
| 0.11 | 9.7% ± 0.66% | 90% ± 0.66% | 0.11 ± 0.0081 | 0.97 ± 0.073 |
|  |  |  |  | Overall: 0.91 ± 0.054 |

**MRM mode in losing H_2_O**

|  | Peak area percentage | |  |  |
| --- | --- | --- | --- | --- |
| [3-^13^C]lactate/lactate  concentration ratio | [3-^13^C]lactate  (*m/z* 90>72) | Lactate  (*m/z* 89>71) | [3-^13^C]lactate/lactate  peak area ratio | [3-^13^C]lactate/lactate peak area ratio  [3-^13^C]lactate/lactate concentration ratio |
| 9.0 | 90% ± 0.21% | 10% ± 0.21% | 9.0 ± 0.21 | 0.99 ± 0.023 |
| 4.0 | 80% ± 0.55% | 20% ± 0.55% | 4.1 ± 0.14 | 1.0 ± 0.035 |
| 2.3 | 71% ± 0.95% | 29% ± 0.95% | 2.5 ± 0.11 | 1.1 ± 0.049 |
| 1.5 | 62% ± 0.57% | 38% ± 0.57% | 1.6 ± 0.039 | 1.1 ± 0.026 |
| 1.0 | 52% ± 0.70% | 48% ± 0.70% | 1.1 ± 0.030 | 1.1 ± 0.030 |
| 0.67 | 42% ± 0.57% | 58% ± 0.57% | 0.73 ± 0.017 | 1.1 ± 0.026 |
| 0.43 | 32% ± 0.45% | 68% ± 0.45% | 0.48 ± 0.010 | 1.1 ± 0.023 |
| 0.25 | 22% ± 0.50% | 78% ± 0.50% | 0.28 ± 0.0081 | 1.1 ± 0.032 |
| 0.11 | 11% ± 0.28% | 89% ± 0.28% | 0.12 ± 0.0034 | 1.1 ± 0.031 |
|  |  |  |  | Overall: 1.1 ± 0.049 |

**Supplementary Table S14.** Standards of [U-^13^C]lactate mixed with lactate, and [U-^13^C]lactate/lactate concentration ratios were: 9.0, 4.0, 2.3, 1.5, 1.0, 0.67, 0.43, 0.25, and 0.11. These mixtures were analyzed by LC-MS/MS under MRM mode in losing CO and losing H_2_O, as well as analyzed by LC-MS under Q1 multiple ion mode.

**MRM mode in losing CO**

|  | Peak area percentage | |  |  |
| --- | --- | --- | --- | --- |
| [U-^13^C]lactate/lactate  concentration ratio | [U-^13^C]lactate  (*m/z* 92>45) | Lactate  (*m/z* 89>43) | [U-^13^C]lactate/lactate  peak area ratio | [U-^13^C]lactate/lactate peak area ratio  [U-^13^C]lactate/lactate concentration ratio |
| 9.0 | 93% ± 0.18% | 7.2% ± 0.18% | 13 ± 0.34 | 1.4 ± 0.038 |
| 4.0 | 85% ± 0.19% | 15% ± 0.19% | 5.7 ± 0.087 | 1.4 ± 0.022 |
| 2.3 | 77% ± 0.29% | 23% ± 0.29% | 3.3 ± 0.054 | 1.4 ± 0.023 |
| 1.5 | 68% ± 0.20% | 32% ± 0.20% | 2.1 ± 0.019 | 1.4 ± 0.013 |
| 1.0 | 59% ± 0.63% | 41% ± 0.63% | 1.4 ± 0.038 | 1.4 ± 0.038 |
| 0.67 | 50% ± 0.20% | 50% ± 0.20% | 0.98 ± 0.0081 | 1.5 ± 0.012 |
| 0.43 | 38% ± 0.62% | 62% ± 0.62% | 0.62 ± 0.012 | 1.5 ± 0.028 |
| 0.25 | 26% ± 0.30% | 74% ± 0.30% | 0.35 ± 0.0055 | 1.4 ± 0.022 |
| 0.11 | 14% ± 0.11% | 86% ± 0.11% | 0.16 ± 0.0015 | 1.4 ± 0.014 |
|  |  |  |  | Overall: 1.4 ± 0.031 |

**Q1 multiple ion mode**

|  | Peak area percentage | |  |  |
| --- | --- | --- | --- | --- |
| [U-^13^C]lactate/lactate  concentration ratio | [U-^13^C]lactate  (*m/z* 92) | Lactate  (*m/z* 89) | [U-^13^C]lactate/lactate  peak area ratio | [U-^13^C]lactate/lactate peak area ratio  [U-^13^C]lactate/lactate concentration ratio |
| 9.0 | 90% ± 0.23% | 10% ± 0.23% | 8.6 ± 0.21 | 0.95 ± 0.024 |
| 4.0 | 79% ± 0.45% | 21% ± 0.45% | 3.8 ± 0.10 | 0.94 ± 0.026 |
| 2.3 | 69% ± 0.61% | 31% ± 0.61% | 2.3 ± 0.064 | 0.97 ± 0.028 |
| 1.5 | 60% ± 0.33% | 40% ± 0.33% | 1.5 ± 0.021 | 1.0 ± 0.014 |
| 1.0 | 50% ± 0.22% | 50% ± 0.22% | 1.0 ± 0.0089 | 1.0 ± 0.0089 |
| 0.67 | 40% ± 0.35% | 60% ± 0.35% | 0.66 ± 0.010 | 0.99 ± 0.015 |
| 0.43 | 30% ± 0.52% | 70% ± 0.52% | 0.42 ± 0.011 | 0.98 ± 0.025 |
| 0.25 | 20% ± 1.1% | 80% ± 1.1% | 0.26 ± 0.018 | 1.0 ± 0.071 |
| 0.11 | 10% ± 0.38% | 90% ± 0.38% | 0.11 ± 0.0030 | 1.0 ± 0.027 |
|  |  |  |  | Overall: 0.99 ± 0.041 |

**MRM mode in losing H_2_O**

|  | Peak area percentage | |  |  |
| --- | --- | --- | --- | --- |
| [U-^13^C]lactate/lactate  concentration ratio | [U-^13^C]lactate  (*m/z* 92>74) | Lactate  (*m/z* 89>71) | [U-^13^C]lactate/lactate  peak area ratio | [U-^13^C]lactate/lactate peak area ratio  [U-^13^C]lactate/lactate concentration ratio |
| 9.0 | 91% ± 0.13% | 8.7% ± 0.13% | 11 ± 0.17 | 1.2 ± 0.019 |
| 4.0 | 82% ± 0.38% | 18% ± 0.38% | 4.7 ± 0.13 | 1.2 ± 0.031 |
| 2.3 | 73% ± 0.57% | 27% ± 0.57% | 2.7 ± 0.078 | 1.1 ± 0.034 |
| 1.5 | 63% ± 0.74% | 37% ± 0.74% | 1.7 ± 0.055 | 1.1 ± 0.036 |
| 1.0 | 54% ± 0.54% | 46% ± 0.54% | 1.2 ± 0.025 | 1.2 ± 0.025 |
| 0.67 | 44% ± 0.27% | 56% ± 0.27% | 0.80 ± 0.0087 | 1.2 ± 0.013 |
| 0.43 | 33% ± 0.60% | 67% ± 0.60% | 0.50 ± 0.014 | 1.2 ± 0.032 |
| 0.25 | 22% ± 0.39% | 78% ± 0.39% | 0.28 ± 0.0065 | 1.1 ± 0.026 |
| 0.11 | 12% ± 0.38% | 88% ± 0.38% | 0.14 ± 0.0049 | 1.3 ± 0.044 |
|  |  |  |  | Overall: 1.2 ± 0.045 |

**Supplementary Table S15. Percentage of lactate isotopomers and isotopologues (mean±SD, n=12) generated by Hela, K562, and thymocytes, traced by [1,2-^13^C_2_]glucose before and after calibration using [2,3-^13^C_2_]lactate.**

|  |  | **Lactate derived from glycolysis** | |  |
| --- | --- | --- | --- | --- |
|  |  | Glc carbon 1,2,3 | Glc carbon 4,5,6 |  |
|  |  | [2,3-^13^C_2_]lactate | lactate | Ratio of [2,3-^13^C_2_]lactate over lactate |
| **Hela** | **Before calibration** | 55% ± 1.1% | 33% ± 0.26% | 1.7 |
|  | **After calibration** | 45% ± 1.5% | 41% ± 1.1% | 1.1 |
| **K562** | **Before calibration** | 56% ± 0.41% | 34% ± 0.64% | 1.6 |
|  | **After calibration** | 46% ± 0.92% | 42% ± 0.76% | 1.1 |
| **Thymocytes** | **Before calibration** | 52% ± 0.48% | 33% ± 0.48% | 1.6 |
|  | **After calibration** | 49% ± 0.48% | 46% ± 0.52% | 1.1 |

The data before calibration are from supplementation Table 2.

**Supplementary Table S16. Percentage of lactate isotopomers and isotopologues (mean±SD, n=12) generated by Hela, K562, and thymocytes, traced by [1-^13^C]glucose before and after calibration using [3-^13^C]lactate.**

|  |  | **Lactate derived from glycolysis** | |  |
| --- | --- | --- | --- | --- |
|  |  | Glc carbon 1,2,3 | Glc carbon 4,5,6 |  |
|  |  | [3-^13^C]lactate | lactate | Ratio of [3-^13^C]lactate over lactate |
| **Hela** | **Before calibration** | 48% ± 0.63% | 36% ± 0.58% | 1.3 |
|  | **After calibration** | 44% ± 0.62% | 40% ± 0.59% | 1.1 |
| **K562** | **Before calibration** | 50% ± 0.32% | 37% ± 0.42% | 1.4 |
|  | **After calibration** | 45% ± 0.32% | 40% ± 0.43% | 1.1 |
| **Thymocytes** | **Before calibration** | 46% ± 0.53% | 36% ± 0.58% | 1.3 |
|  | **After calibration** | 41% ± 0.51% | 39% ± 0.60% | 1.1 |

The data before calibration are from supplementation Supplementary Table S7.

**Supplementary Table S17. Percentage of lactate isotopomers and isotopologues (mean±SD, n=12) generated by Hela, K562, and thymocytes, traced by [6 -^13^C]glucose** **before and after calibration using [3-^13^C]lactate**

|  |  | **Lactate derived from glycolysis** | |  |
| --- | --- | --- | --- | --- |
|  |  | Glc carbon 1,2,3 | Glc carbon 4,5,6 |  |
|  |  | lactate | [3-^13^C]lactate | Ratio of [3-^13^C]lactate over lactate |
| **Hela** | **Before calibration** | 39% ± 0.60% | 46% ± 0.52% | 0.85 |
|  | **After calibration** | 43% ± 0.62% | 42% ± 0.52% | 1.0 |
| **K562** | **Before calibration** | 40% ± 0.30% | 47% ± 0.27% | 0.85 |
|  | **After calibration** | 44% ± 0.31% | 43% ± 0.27% | 1.0 |
| **Thymocytes** | **Before calibration** | 38% ± 0.47% | 44% ± 0.36% | 0.86 |
|  | **After calibration** | 42% ± 0.49% | 40% ± 0.35% | 1.0 |

The data before calibration are from supplementation Supplementary Table S8.

**Supplementary Table S18. Percentage of lactate isotopomers and isotopologues (mean±SD, n=12) generated by Hela, K562, and thymocytes, traced by [1,2,3-^13^C_3_]glucose** **before and after calibration using [U-^13^C]lactate.**

|  |  | **Lactate derived from glycolysis** | |  |
| --- | --- | --- | --- | --- |
|  |  | Glc carbon 1,2,3 | Glc carbon 4,5,6 |  |
|  |  | [U-^13^C]lactate | lactate | Ratio of [U-^13^C]lactate over lactate |
| **Hela** | **Before calibration** | 52% ± 0.56% | 34% ± 0.39% | 1.5 |
|  | **After calibration** | 45% ± 0.56% | 40% ± 0.39% | 1.1 |
| **K562** | **Before calibration** | 53% ± 0.47% | 35% ± 0.53% | 1.5 |
|  | **After calibration** | 45% ± 0.48% | 42% ± 0.57% | 1.1 |
| **Thymocytes** | **Before calibration** | 49% ± 0.33% | 33% ± 0.46% | 1.5 |
|  | **After calibration** | 41% ± 0.32% | 39% ± 0.51% | 1.1 |

The data before calibration are from supplementation Supplementary Table S9
